# Supplementary material for: High-Throughput Assessment of Kinome-wide Activation States
Source: Cell Syst. 2019 Oct 23;9(4):366–374.e5. doi: 10.1016/j.cels.2019.08.005 (PMC6838672; doi:10.1016/j.cels.2019.08.005)
Supplement: Document S1. Figures S1–S5 [file mmc1.pdf]

Cell Systems, Volume *9*

Supplemental Information

High-Throughput Assessment

of Kinome-wide Activation States

Thierry Schmidlin, Donna O. Debets, Charlotte A.G.H. van Gelder, Kelly E. Stecker, Stamatia Rontogianni, Bart L. van den Eshof, Kristel Kemper, Esther H. Lips, Maartje van den Biggelaar, Daniel S. Peeper, Albert J.R. Heck, and Maarten Altelaar

- Supplementary Information –

High-throughput assessment of kinome-wide activation states

Thierry Schmidlin[1,2], Donna O. Debets[1,2], Charlotte A.G.H. van Gelder[1,2], Kelly E. Stecker[1,2], Stamatia Rontogianni[1,2], Bart L. van den Eshof[3], Kristel Kemper[4], Esther H. Lips[5], Maartje van den Biggelaar[3], Daniel S. Peeper[4], Albert J.R. Heck[1,2] and Maarten Altelaar[1,2,6,#,*]

1 Biomolecular Mass Spectrometry and Proteomics, Bijvoet Center for Biomolecular Research and Utrecht Institute for Pharmaceutical Sciences, Utrecht University, Padualaan 8, 3584 CH Utrecht, The Netherlands

2 Netherlands Proteomics Center, Padualaan 8, 3584 CH Utrecht, The Netherlands

3 Sanquin Research, Department of Molecular and Cellular Hemostasis, Amsterdam, the Netherlands.

4 Division of Molecular Oncology, Oncode Institute, The Netherlands Cancer Institute, 1066 CX Amsterdam, the Netherlands

5 Department of Molecular Pathology, The Netherlands Cancer Institute, 1066 CX Amsterdam, the Netherlands.

6 Mass Spectrometry and Proteomics Facility, The Netherlands Cancer Institute, 1066 CX Amsterdam, the Netherlands

[#] To whom correspondence should be addressed: m.altelaar@uu.nl

* Lead contact

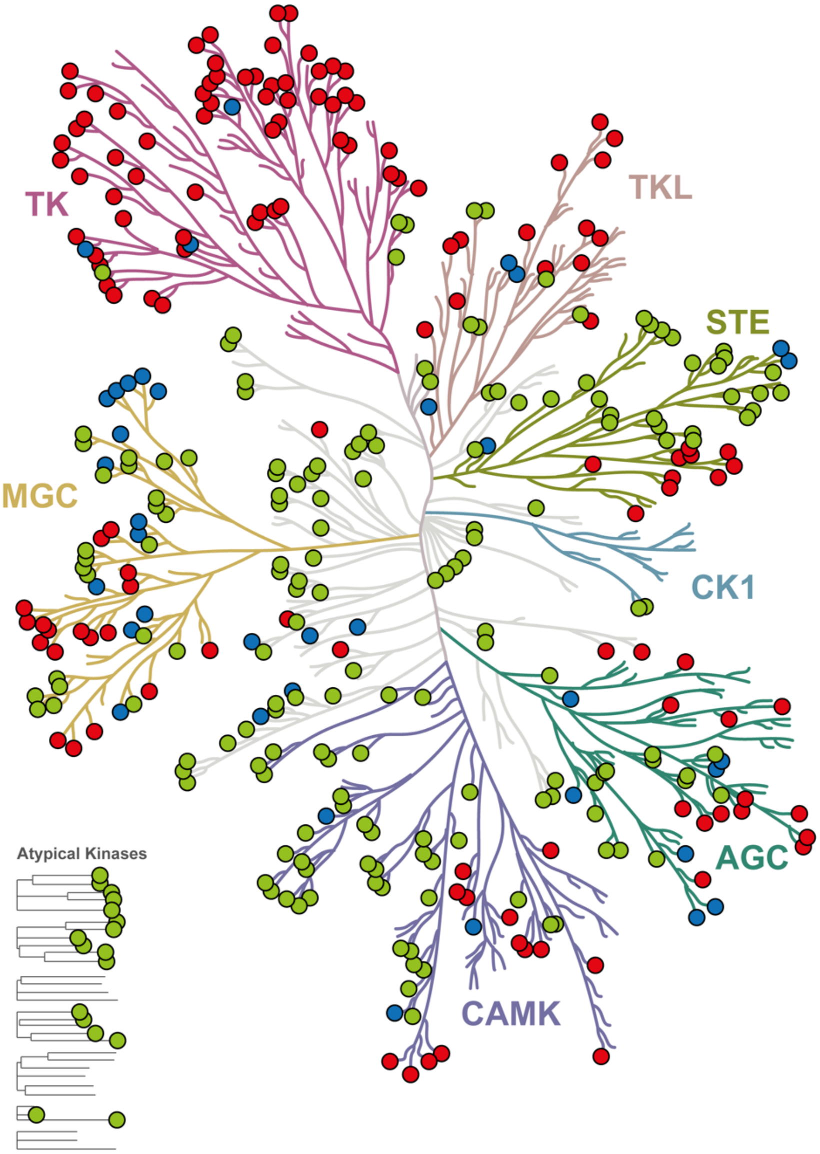

Figure S1, Related to Figure 1: Depiction of the human kinome indicating all kinases currently included in the t-loop screening method (blue circle), kinases being classified as understudied by Collins et al. (green circle) and the overlap of the two (red circles).

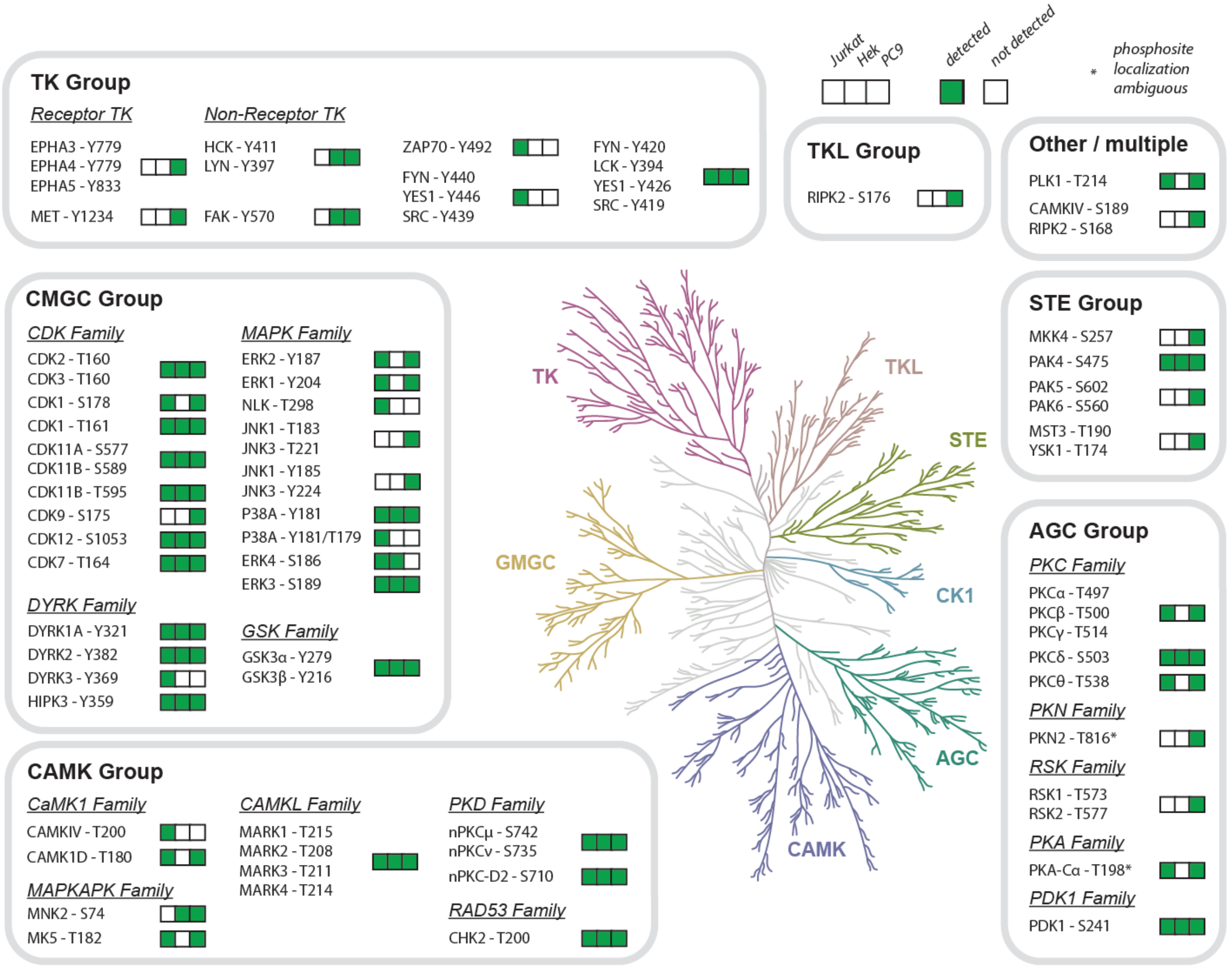

Figure S2, Related to Figure 1: Compendium of t-loop phosphorylations detected in Jurkat, HEK293T and PC9 cell lines cultured under standard conditions without any form of stimulation.

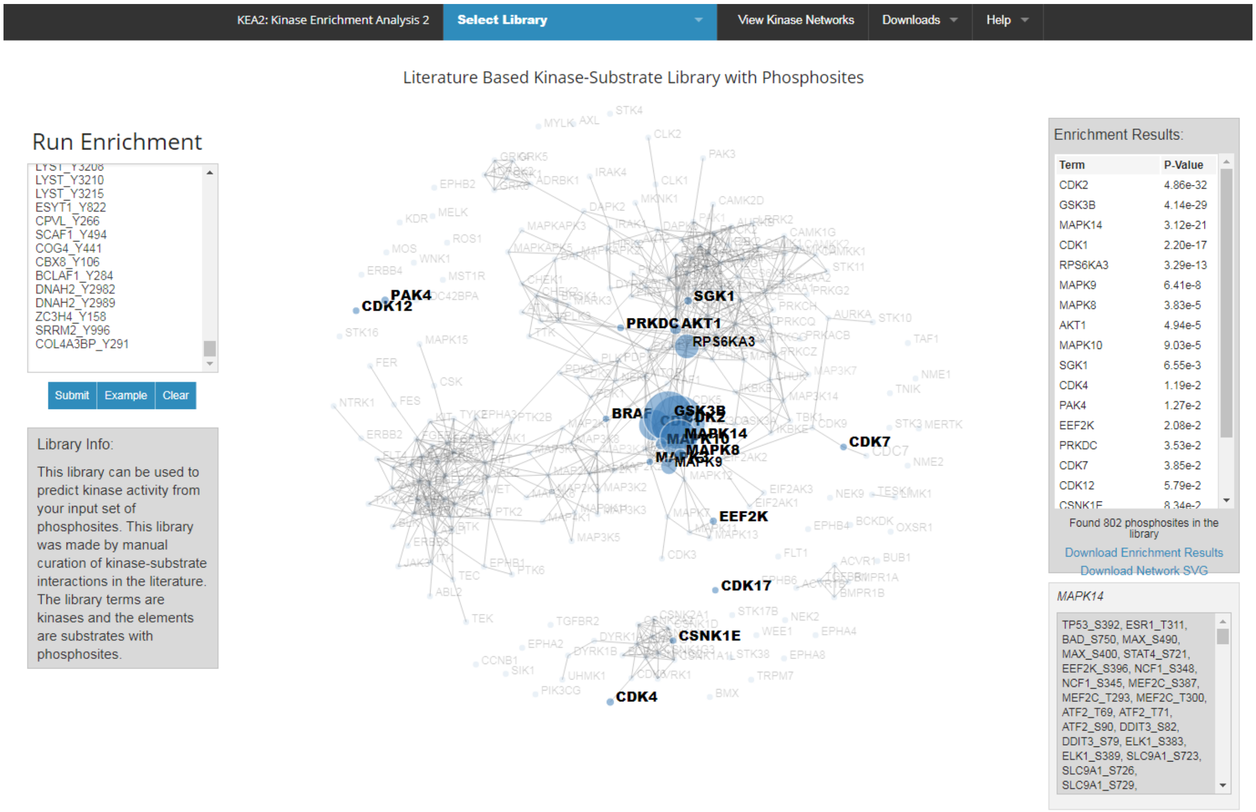

Figure S3, Related to Figure 2: Screenshot of the KEA2 output reporting kinase activity determined from Jurkat cell lysates subjected to high-pH reversed phase fractionation and subsequent phosphopeptide enrichment analyzed by shotgun LC-MS.

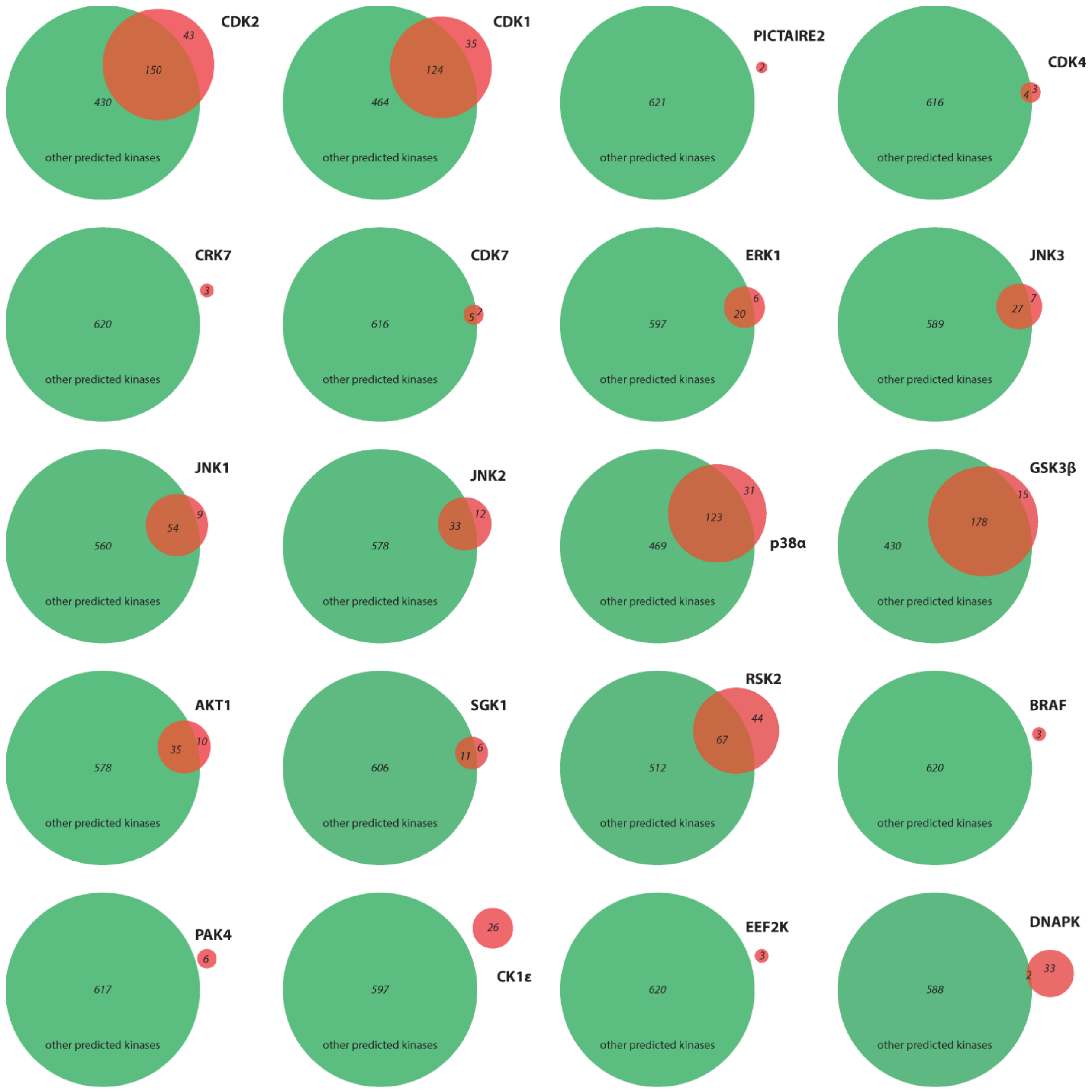

Figure S4, Related to Figure 2: Venn diagrams illustrating the amount of redundancy in kinase-substrate relationships impeding the kinase prediction by the online tool KEA2. Each Venn diagram compares the reported substrates to the reported substrates of the other predicted kinases.

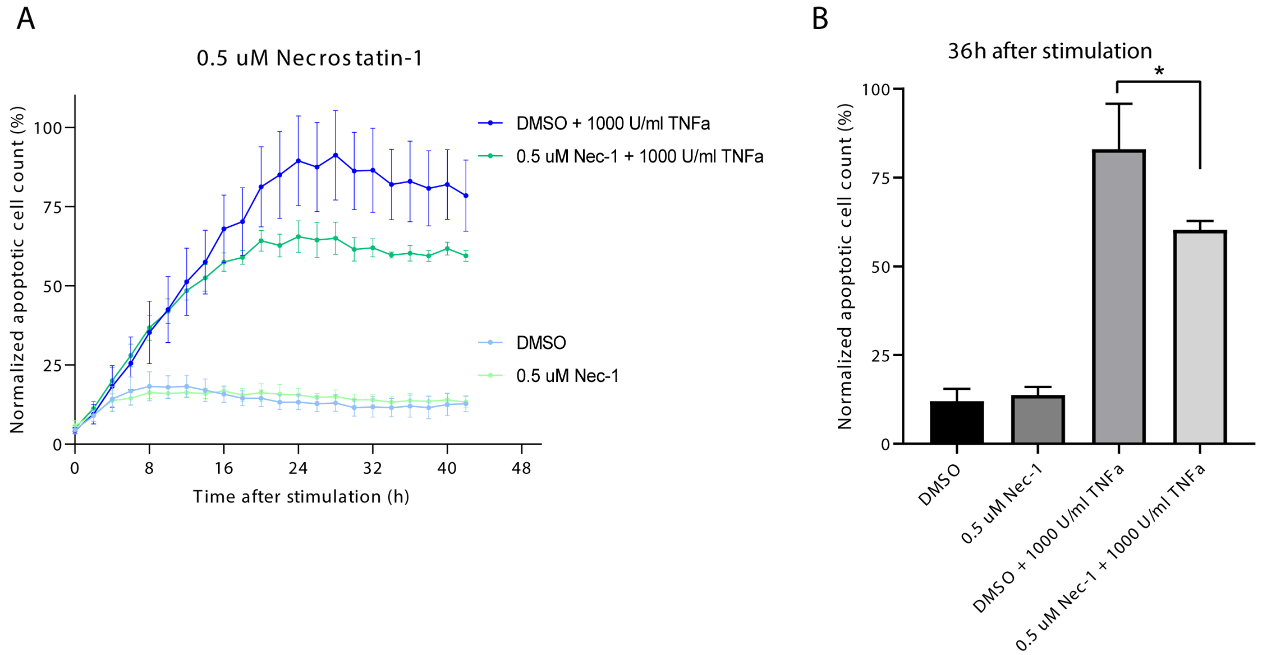

Figure S5, Related to Figure 2: Jurkat cell apoptosis upon TNFα treatment: Jurkat cells were grown with and without TNFα stimulation, in presence or absence of necrostatin-1 (Nec-1), and rate of cell death was determined by Caspase-3/7 green apoptosis reagent (n = 4 for all groups, error bars depicting standard deviation). (B) Apoptotic cell count after 36 hours in the different conditions showing a significant reduction in apoptotic cells after TNFα stimulation in the presence of Nec-1.
