# Supplementary material for: High-Throughput Assessment of Kinome-wide Activation States
Source: Cell Syst. 2019 Oct 23;9(4):366–374.e5. doi: 10.1016/j.cels.2019.08.005 (PMC6838672; doi:10.1016/j.cels.2019.08.005)
Supplement: Document S2. Article plus Supplemental Information [file mmc10.pdf]

Cell Systems

Report

High-Throughput Assessment of Kinome-wide Activation States

Graphical Abstract

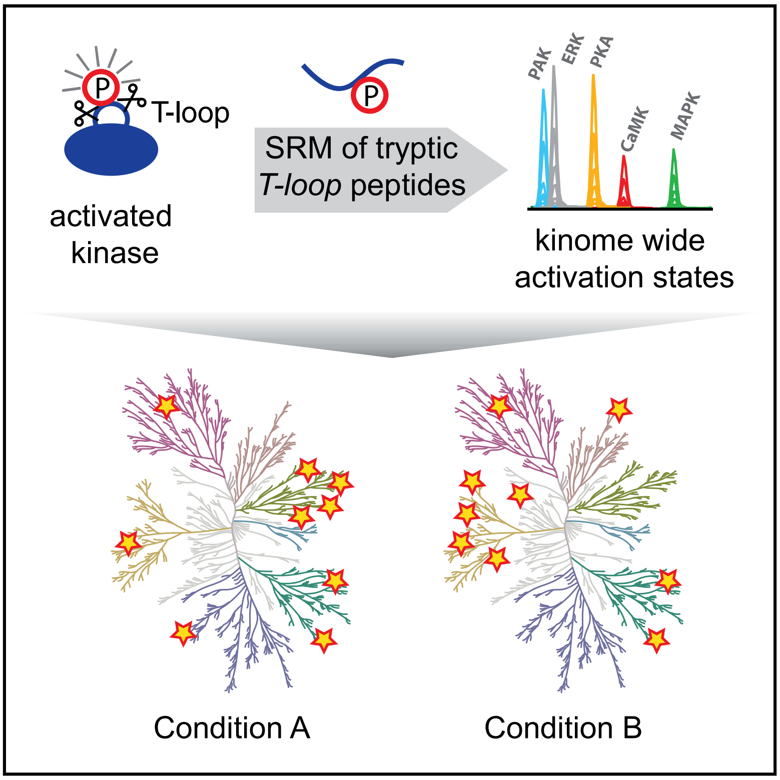

Highlights

- Robust targeted MS assays permit observation of conserved kinome activation sites
- 178 human kinases are characterized in high-throughput assays
- Kinase activation states are observed in human primary cells and needle biopsy
- Specific kinase activation states are induced during cell death and drug resistance

Authors

Thierry Schmidlin, Donna O. Debets, Charlotte A.G.H. van Gelder, ..., Daniel S. Peeper, Albert J.R. Heck, Maarten Altelaar

Correspondence

m.altelaar@uu.nl

In Brief

We introduce a method for rapid screening of system-wide kinase activation states. Our approach relies on targeted quantification of evolutionary conserved activator sites, allowing the omission of the current bias toward well-studied kinases with known kinase-substrate relationships. Universally applicable to all types of human samples, the method is characterized by high sensitivity and throughput, providing extensive coverage of the human kinome, uncovering insights into kinase activation states in various molecular processes.

Schmidlin et al., 2019, Cell Systems *9*, 366–374
October 23, 2019 © 2019 The Author(s). Published by Elsevier Inc.
https://doi.org/10.1016/j.cels.2019.08.005

OPEN ACCESS
CellPress

Cell Systems
Report

High-Throughput Assessment of Kinome-wide Activation States

Thierry Schmidlin,[1,2] Donna O. Debets,[1,2] Charlotte A.G.H. van Gelder,[1,2] Kelly E. Stecker,[1,2] Stamatia Rontogianni,[1,2] Bart L. van den Eshof,[3] Kristel Kemper,[4] Esther H. Lips,[5] Maartje van den Biggelaar,[3] Daniel S. Peeper,[4] Albert J.R. Heck,[1,2] and Maarten Altelaar[1,2,6,7,*]

[1]Biomolecular Mass Spectrometry and Proteomics, Bijvoet Center for Biomolecular Research and Utrecht Institute for Pharmaceutical Sciences, Utrecht University, Padualaan 8, 3584 CH Utrecht, the Netherlands
[2]Netherlands Proteomics Center, Padualaan 8, 3584 CH Utrecht, the Netherlands
[3]Sanquin Research, Department of Molecular and Cellular Hemostasis, Amsterdam, the Netherlands
[4]Division of Molecular Oncology, Oncode Institute, The Netherlands Cancer Institute, 1066 CX Amsterdam, the Netherlands
[5]Department of Molecular Pathology, The Netherlands Cancer Institute, 1066 CX Amsterdam, the Netherlands
[6]Mass Spectrometry and Proteomics Facility, The Netherlands Cancer Institute, 1066 CX Amsterdam, the Netherlands
[7]Lead Contact
*Correspondence: m.altelaar@uu.nl
https://doi.org/10.1016/j.cels.2019.08.005

SUMMARY

Aberrant kinase activity has been linked to a variety of disorders; however, methods to probe kinase activation states in cells have been lacking. Until now, kinase activity has mainly been deduced from either protein expression or substrate phosphorylation levels. Here, we describe a strategy to directly infer kinase activation through targeted quantification of T-loop phosphorylation, which serves as a critical activation switch in a majority of protein kinases. Combining selective phosphopeptide enrichment with robust targeted mass spectrometry, we provide highly specific assays for 248 peptides, covering 221 phosphosites in the T-loop region of 178 human kinases. Using these assays, we monitored the activation of 63 kinases through 73 T-loop phosphosites across different cell types, primary cells, and patient-derived tissue material. The sensitivity of our assays is highlighted by the reproducible detection of TNF-α-induced RIPK1 activation and the detection of 46 T-loop phosphorylation sites from a breast tumor needle biopsy.

INTRODUCTION

Kinases are key regulators of inter- and intracellular communication, and their inhibitors are critical in targeted therapy and precision medicine (Blume-Jensen and Hunter, 2001; Garay and Gray, 2012; Lahiry et al., 2010). Therefore, the capability to monitor the dynamics of kinase activity is essential to deepen our understanding of cellular function and could greatly impact rational drug design. Since the complete cataloging of all human kinases by Manning et al. (2002), an increasing number of studies have focused on the so-called "kinome;" however, robust methods to determine kinase activation on a kinome-wide level are still lacking.

Initial attempts to measure global kinase activation states exploited the interaction of kinases with immobilized unspecific multiplexed inhibitor beads (MIBs), which demonstrated altered binding affinities upon enzymatic activation (Bantscheff et al., 2007). This primed various studies to interpret increased MIB binding affinity as increased kinase activity on a kinome-wide scale (Stuhlmiller et al., 2015, 2017). However, it was later shown that MIBs bind kinases largely independent of their activation status (Ruprecht et al., 2015). In line with this approach, several studies reported the use of biotin-conjugated acyl-nucleotide probes for the enrichment of kinases via their ATP-binding pocket from complex backgrounds (Patricelli et al., 2011; Xiao et al., 2014). In combination with targeted mass spectrometry (MS), these methods allow quantification of the expression of >200 kinases; however, they do not directly deduce kinase activity.

Alternative approaches to assess kinome activity involve analyzing large-scale phosphoproteomics datasets for over- or underrepresented sequence motifs that can be linked to known kinase substrates or sequence specificities (Lachmann and Ma'ayan, 2009; Miller et al., 2008). Indeed, we and others have utilized such prediction tools to infer kinase activity profiles (Guo et al., 2011; Zagorac et al., 2018); however, these methods severely suffer in sensitivity due to lack of knowledge on the majority of kinase substrates and thus display a strong bias toward well-studied kinases (Ding et al., 2011). Tackling these limitations requires the analysis of direct markers for kinase activation.

The majority of protein kinases are regulated through phosphorylation of their activation loop (T-loop). The well-studied functionality of these phosphorylations, in combination with their high level of evolutionary conservation throughout the entire kinome, facilitates the use of T-loop phosphorylation as a direct probe to assess kinase activation states (Nolen et al., 2004). In principle, T-loop phosphorylation can be detected using phospho-specific antibodies, but their availability is limited to a few well-studied kinases. Furthermore, T-loop phosphorylations often remain undetected or cannot be accurately quantified in large-scale phosphoproteomics studies due to the low abundance of the corresponding peptides.

Sensitive and accurate characterization of selected phosphorylation events may be achieved by targeted MS approaches.

 © 2019 The Author(s). Published by Elsevier Inc.
This is an open access article under the CC BY license (http://creativecommons.org/licenses/by/4.0/).

Previous studies have shown the potential of using targeted quantification of proteins or post-translational modifications (PTMs) as a highly specific readout for previously characterized biological functions in yeast (Soste et al., 2014) or human samples (Abelin et al., 2016). However, both previous approaches heavily rely on diverse well-characterized or at least empirically established cellular characteristics (e.g., starvation and proliferation).

Here, we present a targeted MS method for the system-wide quantification of kinase T-loop phosphorylation levels, providing a direct and unbiased readout of cellular kinase activation states. Specifically, our approach combines selected reaction monitoring (SRM)-MS with highly specific Fe(III)-immobilized metal affinity chromatography (IMAC) phosphopeptide enrichment on an automated platform with parallel phosphopeptide enrichment of up to 96 samples. We demonstrate the general applicability of our strategy by analyzing the activation profiles of 178 kinases in various cell lines, blood platelets, and a breast cancer biopsy sample. Our approach allows rapid assessment of both well-characterized and novel biological processes, ranging from high-throughput diagnosis up to hypothesis-free screening experiments.

RESULTS

Our method currently encompasses robust molecular assays to accurately quantify 221 phosphorylation sites in the T-loop region of 178 kinases (Figure 1A; Table S1). This number is primarily based on accessibility of the T-loop phosphopeptides by trypsin and can be expanded by the use of alternative proteases. Here, the reported assays represent roughly one-third of the human kinome (Manning et al., 2002) and will be made available via Table S2 and are uploaded in Peptide Atlas (Desiere et al., 2006). Key challenges associated with the detection of T-loop phosphorylation in shotgun data sets such as low abundance, unfavorable liquid chromatography (LC)-MS characteristics, and a high prevalence of tyrosine phosphorylations, have been largely solved by exploiting the combination of high-specificity phosphopeptide enrichment with the unparalleled sensitivity of nanoLC-SRM on a triple-quadrupole MS. All assays are available in a survey and a quantification mode (Figures 1B and 1C). The survey mode allows for a rapid screening for activation states of all 178 kinases in a 2 h analysis, while the quantification mode enables accurate quantification of those kinases observed in the survey scan and includes confident phosphosite localization and a high tolerance for interferences.

To gain insight into the sensitivity of the method, we measured a dilution series of >40 synthetic T-loop peptides spiked into a background of phosphopeptides enriched from a tryptic whole-cell digest. The resulting linear calibrations curves were used to determine the limits of quantification (LOQ) and detection (LOD), ranging from 0.2 to 10 fmol for the LOD and 0.7 to 34 fmol for the LOQ (Figure 1D; Table S3). Important to mention here is that several kinase T-loops can become doubly phosphorylated, resulting in doubly phosphorylated peptides after tryptic digestion. In current proteomics workflows, detection of these doubly phosphorylated peptides can be hampered because of poor ionization efficiency and inefficient elution after phosphopeptide enrichment. Our final list of targeted kinases comprise clinically relevant kinases with inhibitors approved by the Food and Drug Administration (FDA) such as Met, Abl, Src, BTK, Jak3, and Kit (Fabbro et al., 2015) (Table S4) as well as numerous kinases classified as understudied (Collins et al., 2018) (Figure S1).

As a first assessment, we analyzed the baseline kinome activation state of three different human cell lines: (1) Jurkat cells, an immortalized line of T lymphocytes; (2) PC9 cells, a non-small cell lung cancer cell line; and (3) HEK293T cells, a cell line derived from human embryonic kidney cells. Without any form of stimulation, we were able to cumulatively detect 52 T-loop phosphorylation sites (Figure S2). Because of the highly conserved nature of the kinases' T-loop sequence, the representative tryptic peptides are not always unique. To deal with this ambiguity, we followed the principle of protein grouping (Nesvizhskii and Aebersold, 2005) and refer to these instances as kinase groups throughout this study. For the 52 phosphorylation sites observed, this resulted in 48 kinase groups (Table S5). Unsurprisingly, a large part of the 48 detected kinase groups represented kinases crucial for cell growth under typical culturing conditions, such as cyclin-dependent kinases (CDKs) and mitogen-activated protein kinases (MAPKs) as well as the two abundant kinases PDK1 and GSK3.

It is noteworthy that various kinases show cell-type-specific activation states. For instance, both Jurkat and PC9 cells showed an increased activity of Ca^{2+} and diacylglycerol (DAG)-dependent signaling compared to HEK293T cells, with several kinases from the Ca^{2+}/calmodulin-dependent protein kinase (CaMK) group and the protein kinase C (PKC) family being detected in their active state. PC9 cells show increased activation of tyrosine kinase family members such as FAK, Met, and the two kinase groups ephrin type-A receptors 3, 4, and 5 (EphA3-4-5) and HCK-Lyn, likely due to elevated tyrosine kinase signaling via increased EGFR activity (Sharifnia et al., 2014). Zap70, on the other hand represents a highly tissue-specific kinase, exclusively expressed in cell types associated with the immune system, including T cells. Accordingly, here, it was exclusively detected in Jurkat cells, confirming the specificity of our approach.

To benchmark our approach against two established online tools to predict kinase activity, NetworKIN (Miller et al., 2008) and KEA2 (Lachmann and Ma'ayan, 2009), we performed a deep phosphoproteomic shotgun experiment on Jurkat cell lysates detecting a total of >11,600 phosphorylation sites. Both algorithms rely on detecting substrate motifs or known kinase substrates in large shotgun phosphoproteomic experiments, and Figure 2A depicts NetworKIN scores and KEA2 enrichment results in comparison to abundance values obtained from our targeted T-loop assay (Table S6). Combined, both algorithms predicted activity of 18 out of the 31 kinases measured in our targeted kinase assay, performed using a single nanoLC-MS run on Jurkat cells, confirming the activity of kinases such as GSK3, PKC, PAK4, CDK1, or ERK1/2. As expected, a large number of kinases were exclusive to our targeted approach demonstrating the higher sensitivity of our method. The absence of kinase activity prediction for numerous kinases by both algorithms can be attributed to the lack of knowledge on kinase-substrate relations. This causes the prediction tools to be intrinsically biased toward well-characterized kinases, neglecting the majority of phosphorylation sites (KEA2 incorporated only 800 out of 11,600 phosphosites for the enrichment analysis) (Figure S3). In addition, both prediction tools often disagree, reducing the reliability of the predicted kinase activations.

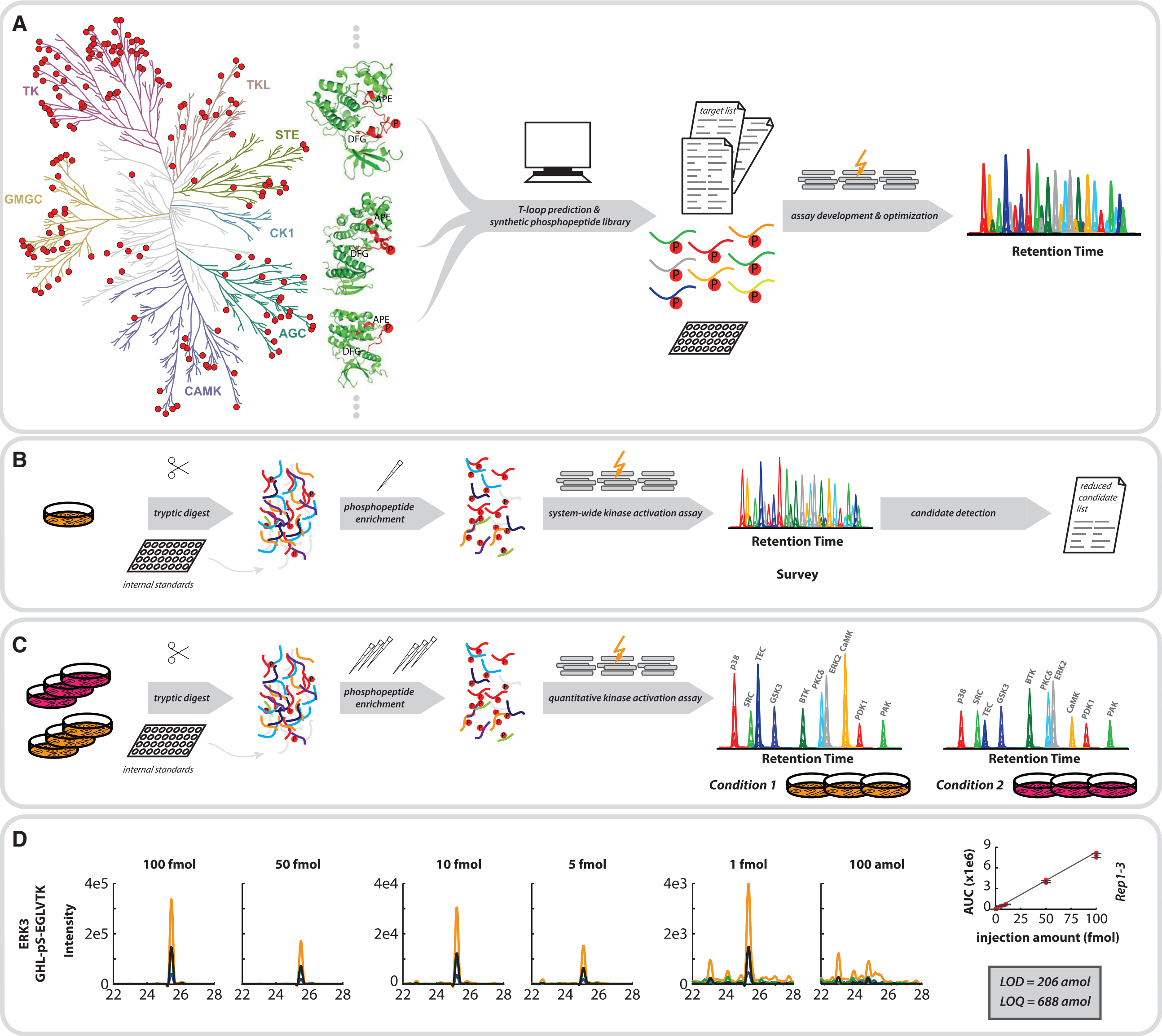

Figure 1. Targeted Quantification of T-Loop Phosphorylation to Determine Kinase Activation

(A) Conserved activation sites were determined based on sequence homologies on a kinome-wide scale. A target list was developed containing phosphorylation sites accessible by tryptic digest covering 33% of the human kinome. Heavy-isotope-labeled peptides were synthesized for all entries of the target list and used for nanoLC-SRM assay development.

(B and C) Samples were subjected to tryptic digest and automated phosphopeptide enrichment using Fe(III)-IMAC on an Agilent Bravo AssayMap. Heavy-isotope-labeled synthetic phosphopeptides were used as internal standards. (B) The survey mode allows screening for kinase activation states for all 178 kinases in one LC-MS run using a limited number of transitions per peptide. (C) The quantification mode employs more transitions per peptide allowing for accurate quantification of kinase activation states across different conditions, including confident phosphosites localization and high tolerance to interferences.

(D) LOD and LOQ values were determined for a subset of phosphopeptides exemplified for the peptide GHL-pS-EGLVTK, representing ERK3. Response rates of the phosphopeptide were determined within a representative phosphopeptide background for known concentrations in triplicate. Variations in the linear regressions were then used to determine LOD and LOQ values according to the formulas LOD = $3S_a/b$ and LOQ = $10S_a/b$ (b = slope and S_a = standard deviation of the intercept). Error bars represent standard deviation observed for triplicate measurements.

Through direct monitoring of T-loop phosphorylations, we were able to alleviate this bias resulting in the detection of numerous understudied kinases in their activated state. Of note, activity for several kinases predicted by KEA2 or NetworKIN are thus far not included in our T-loop assays, a shortcoming that can be overcome by expanding the set of kinases in our approach using alternative proteases. A few kinases reported by the prediction tools were included in our assays but not detected (e.g., RSK2, JNK1, JNK2, and Braf), which could be caused by differences in timing between T-loop versus substrate phosphorylation or, more likely, redundancies in kinase-substrate relations, resulting in false positive predictions as exemplified in Figure S4.

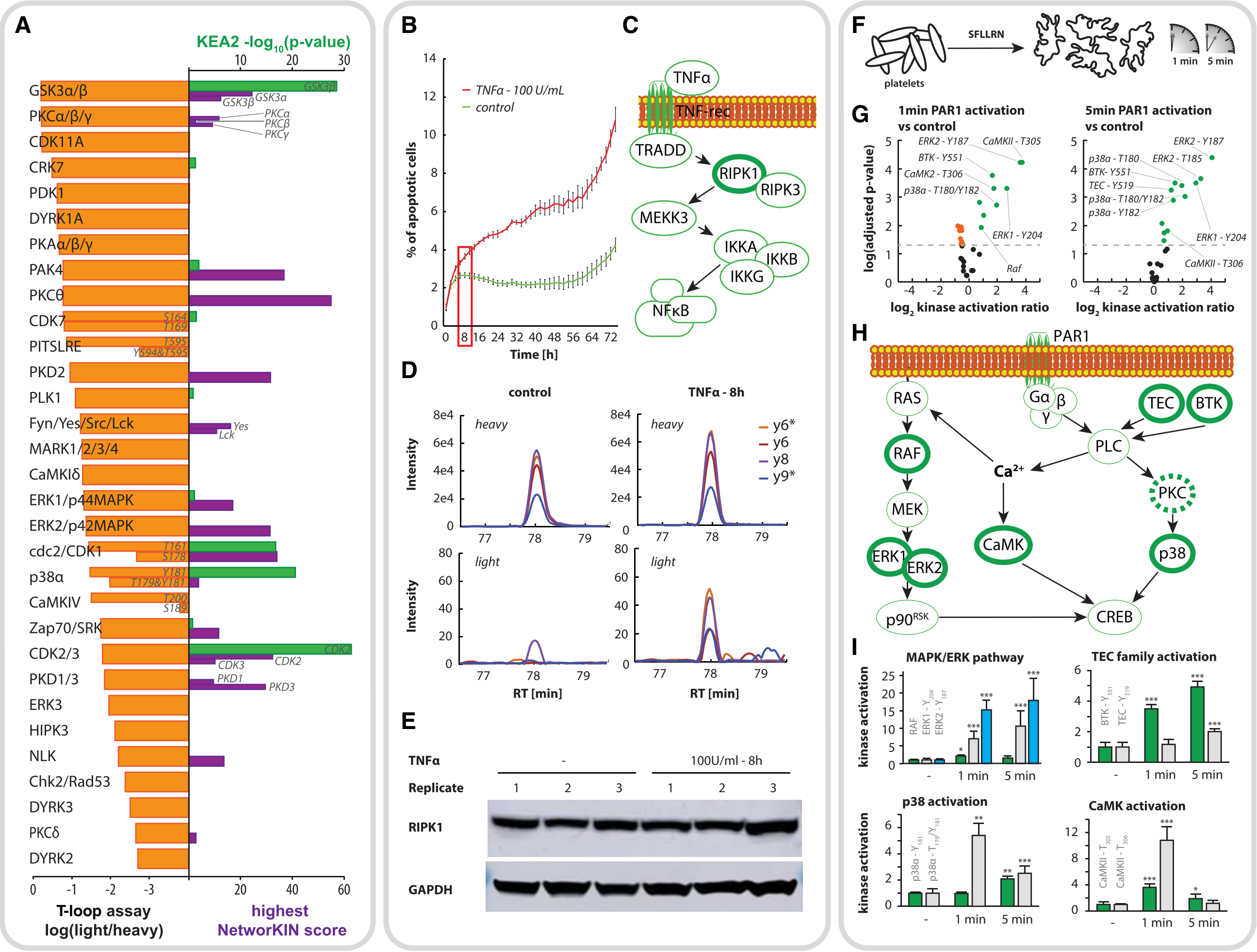

Figure 2. Probing Kinase Activation in Varying Cellular Systems

(A) Determining kinase activation by targeted measurement of T-loop phosphorylations versus modeling of kinase activities from substrate detection in large shotgun proteomic datasets: orange bars represent the relative abundance of T-loop phosphorylations in Jurkat cells estimated by their intensity ratio to the heavy-isotope-labeled standard peptides; green and purple bars represent kinase activity prediction by KEA2 (result as p values, green bars) and NetworKIN (depicted as highest individual score found for any substrate, purple bars) when applied to a fractionated Jurkat cell sample analyzed in shotgun MS (>11,600 phosphopeptides).

(B) Alterations in kinase activation upon TNF-α treatment: Jurkat cells were grown with and without TNF-α stimulation for 8 h, resulting in an increased rate of cell death as determined by caspase-3/7 green apoptosis reagent (n = 4 for both groups, error bars depicting standard deviation).

(C) TNF-α induces the recruitment of receptor-interacting protein serine-threonine kinases (RIPKs) to the TNF-receptor complex, resulting in its activation and initiation of necroptotic signaling.

(D) RIPK1 activation through phosphorylation at S161 measured by SRM under both conditions. Representative SRM traces are shown for unstimulated and stimulated cells with upper panels representing internal heavy-labeled standard peptides and lower panels representing signals from endogenous peptides (* indicates neutral loss of phosphoric acid on the fragment ions).

(E) Immunoblot analysis demonstrates equal protein expression levels of RIPK1 under both conditions.

(F) Kinase activity profiling in PAR1-activated human blood platelets: platelets were activated with SFLLRN-NH_2 hexapeptide, mimicking thrombin activation for 1 and 5 min, respectively.

(G) Volcano plots depict changes in overall kinase activation upon PAR1 activation of platelets for 1 and 5 min, respectively (significance cutoff $p < 0.05$).

(H) Signaling network depicts known downstream signaling pathways of PAR1, highlighting key players where increased T-loop phosphorylation could be monitored in our assay (solid bold green). Baseline PKC activity could be detected for various PKC isozymes but with no significant change in activation upon PAR1 activation (outlined in dashed bold green).

(I) Bar graphs show representative dynamic regulation of kinase activation for key players in the pathway, i.e., p38α, CaMK2, RAF, ERK, BTK, and TEC (quantification based on heavy-to-light ratio, normalized on Ctrl sample, error bars depict standard deviation, *$p \leq 0.05$; **$p \leq 0.01$; ***$p \leq 0.001$).

After the successful detection of several T-loop phosphorylations in unstimulated cells, we reasoned that our technique should be able to reveal activation of specific kinases from the steady-state background upon selected stimuli. To demonstrate this capacity, we treated Jurkat cells with TNF-α for 8 h, which resulted in increased cell death (Figure 2B). Upon TNF-α stimulation, the receptor-interacting protein serine-threonine kinase (RIPK) is recruited to the TNF-receptor complex and mediates

apoptosis and/or necroptosis (Annibaldi and Meier, 2018; Holler et al., 2000) (Figure 2C). Indeed, our method was able to reproducibly detect RIPK1 phosphorylation at S161 already upon TNF-α treatment for 8 h, a phosphorylation not detectable in untreated Jurkat cells (Figure 2D). Western blot analysis for RIPK1 shows no discernible change across the two conditions at the protein expression level (Figure 2E), advocating RIPK1 activation via its T-loop phosphorylation to mediate apoptosis. Inhibition of RIPK1 activity by its inhibitor necrostatin-1 (Nec-1), resulted in reduction of the number of apoptotic cells after 36 h (Figure S5), highlighting the functional relevance of the observed kinase activation.

Remarkably, despite the well-characterized role of RIPK1 in cell death, to the best of our knowledge this represents the first direct detection by MS of RIPK1 T-loop phosphorylation from cell lysates. Thus far, the only report of successful MS detection of phosphorylated S161 in RIPK1 was described by Degterev et al. (2008) using a protein expression system in combination with IP, *in vitro* kinase assay, and phosphopeptide enrichment. This lack of evidence for RIPK1 activation in the literature primed us to further investigate its detectability in shotgun MS. Indeed, even performing a large-scale phosphoproteomics experiment, including high-pH fractionation, did not enable detection of RIPK1 phosphorylation at S161 among the >11,600 detected phosphopeptides. Hence, our targeted approach offers a so far unachieved sensitivity in measuring S161 RIPK1 activation upon TNF-α signaling, providing an additional valuable tool to monitor the complex regulation of cell death.

Next, we wanted to exploit the sensitivity of our method, performing in-depth analyses of rapid kinome dynamics in primary human cells. We applied our technique to study the mechanism of PAR1-mediated activation of blood platelets (Figure 2F). Platelet activation involves various intracellular signaling events; however, the key step is activation of Phospholipase C (PLC), resulting in an increase in intracellular Ca^{2+}. This in turn activates PKC and CaMK signaling and results in activation of RAS, via its translocation to the plasma membrane, which subsequently activates the MAPK cascade (Grover et al., 2018). By performing PAR1 activation for 1 and 5 min, we were able to closely monitor changes in kinase activation states. Overall, we were able to detect and quantify 32 T-loop phosphorylations in 27 kinase groups (Figure 2G), including major players of both PKC and CaMK signaling and the MAPK cascade (Figure 2H; Tables S5 and S7). The well-established nature of the signaling cascade in combination with the two time points additionally allowed us to determine interesting basic signaling kinetics (Figure 2I) hinting toward a rapid response by p38 and CaMK signaling upon PAR1 activation, compared to a slower response by the RAF-MEK-ERK cascade.

Lastly, our assay allowed us to study activation dynamics of the two TEC family tyrosine kinases BTK and TEC, both known to act as major PLCγ2 activators upon platelet activation. Both show an increase in T-loop phosphorylation upon platelet activation; however, BTK seems to be activated faster and to a larger extend, corroborating its leading role over TEC established in the literature (Atkinson et al., 2003).

Since kinases are a major class of drug targets, especially in cancer where 25 kinase-targeting drugs have been approved and numerous candidates are under clinical evaluation (Gross et al., 2015), we wanted to assess the usefulness of our technology to study unbalanced activity of kinases in disease. A major challenge in kinase inhibitor treatment is the (long-term or downstream) effect on the rest of the kinome, which consistently leads to therapy resistance due to adaptation of cellular signaling networks. To demonstrate the potential of our technology to shed light on such mechanisms, we next probed kinase activation upon acquired BRAF inhibitor (BRAFi) resistance in melanoma. Roughly half of all melanomas are driven by the $BRAF^{V600E}$ mutation, resulting in constitutive activity of BRAF kinase activity. Patient treatment with BRAFi shows initial success, but commonly the clinical benefit is only transient because of rapid acquisition of drug resistance (Wagle et al., 2011). Here, we exploit matched patient-derived melanoma cell lines from treatment-naive, treatment-sensitive, and $NRAS^{Q61K}$-based resistant tumor states established from patient-derived tumor xenografts to study BRAF-resistance-driven alterations in kinase activation states (Figure 3A) (Kemper et al., 2015). Across all three cell lines, we were able to detect and quantify 39 phosphosites representing T-loop phosphorylations of 37 kinase groups (Table S5). Several of the quantified kinases showed increased activation in the resistant cell line compared to the treatment-naive and sensitive cells (Figure 3B; Table S7). Many of them are known to be involved in growth and proliferation such as ERK2, MAP2K4, CDK2/CDK3 and NLK, and various members of the PKC kinase family. Surprisingly, some kinases specifically activated in the drug resistant cell line have thus far mainly been linked to tumor suppressing activities such as Chk2 and p38α (Wagner and Nebreda, 2009).

The most striking activation we observed was that of the kinase ERK4, which was around or below the detection limit in the sensitive and naive cell lines, however, was found to be strongly activated (~30 times) upon acquired drug resistance (Figure 3C). This strong activation remained in resistant cells upon drug withdrawal. It has been previously observed that oncogenic RAS increases RNA levels of ERK4 (Kostenko et al., 2012), although no effect on kinase activation level has been reported so far. Our results, however, convey the use of ERK4 activation as a potential marker for NRAS-mediated drug resistance.

The clinical value of our technology ultimately depends on its direct applicability in primary tissue samples, such as tumor biopsies. Here, we demonstrate the sensitivity of our approach by quantifying kinase activation states in a Her2+ breast cancer patient, analyzing tissue material obtained from half a 14G needle biopsy taken prior to treatment (Figure 3D). Using a starting amount of merely 300 μg of protein, we were able to detect and quantify 46 phosphorylation sites in the T-loop region of 43 kinase groups, spanning a dynamic range of more than 3 orders of magnitude (Figure 3E; Table S5). The detected kinases include, besides a substantial number of understudied kinases such as CDK11A, CRK7, DYRK1A, DYRK2, DYRK4, HIPK3, NEK6, and PKN2 (Collins et al., 2018), various crucial players in control of growth and proliferation (Figure 3F), a variety of which are targets for potent novel inhibitors currently in clinical trials. This includes detection of cdc2/CDK1 and CDK2, which can be inhibited by Dinaciclib and ERK1 and ERK2, the phosphorylation of which can be inhibited by various MEK inhibitors such as Selumetinib. Both inhibitors are currently in phase III

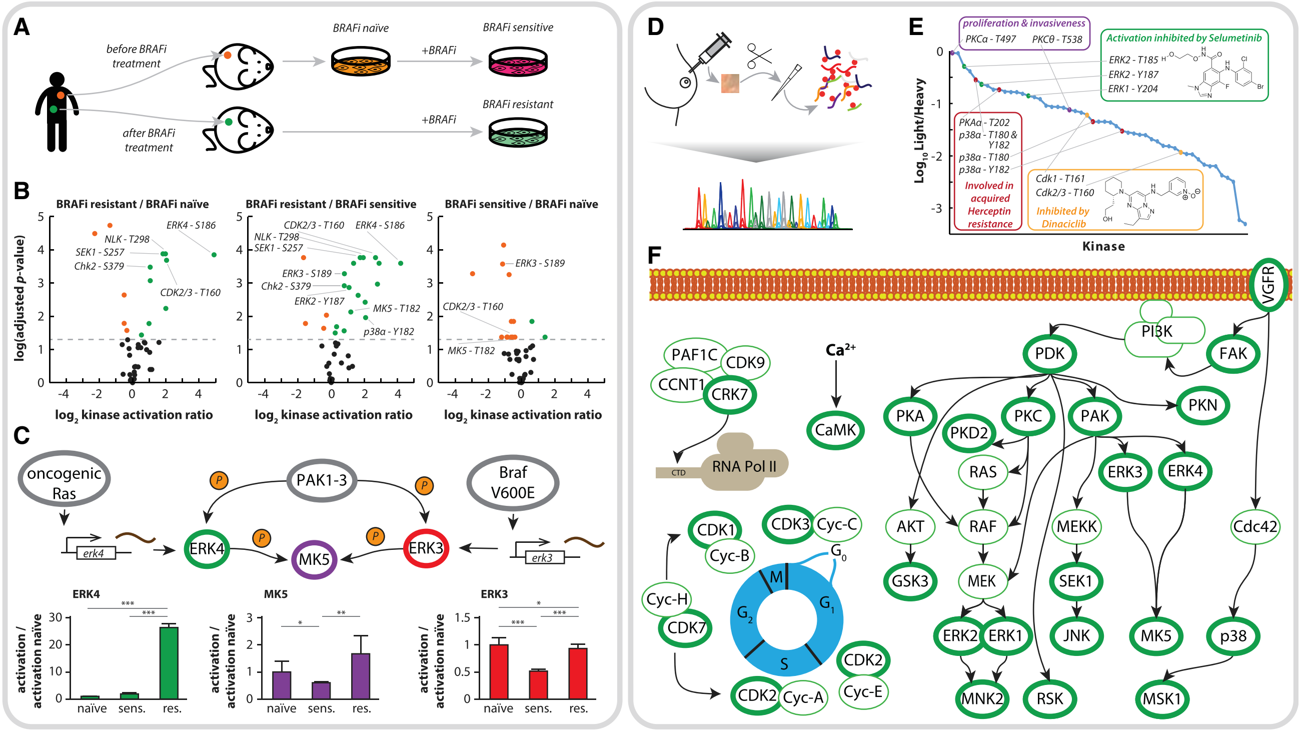

Figure 3. Probing Kinase Activation in Patient-Derived Samples

(A) Reorganization of kinase activities upon acquired BRAF inhibitor resistance in melanoma: matched melanoma cell lines were established from patient-derived xenografts (PDXs) from the same patient before and after acquired resistance to the BRAF inhibitor PLX-4720, giving rise to a model system comprising treatment naive, treatment sensitive, and treatment resistant cell lines.

(B) Pairwise comparison of kinase activation between the 3 conditions is depicted as volcano plot using an arbitrary significance cutoff ($p < 0.05$).

(C) Molecular function of ERK4, which is part of a highly confined molecular system, together with ERK3 and MK5. Bar plots show the detected abundance of the T-loop phosphorylations for ERK3, ERK4, and MK5 (normalized to values observed in naive cells, error bars depicting standard deviation, $^{*}p \leq 0.05$; $^{**}p \leq 0.01$; $^{***}p \leq 0.001$).

(D) Determining kinase activation states in a breast cancer needle biopsy: proteins were extracted from half a needle biopsy (<5 mg of tissue), followed by tryptic digest, phosphopeptide enrichment by Fe(III)-IMAC, and global kinase activation screening by nanoLC-SRM.

(E) The detected kinases span a dynamic range of more than 3 orders of magnitude, as estimated by their intensity ratio to the heavy-isotope-labeled standard peptide (assuming roughly equimolar internal standard concentration).

(F) Selected kinases detected in their activated state are shown in the context of their molecular signaling pathway according to the Kegg database (outlined in bold green).

trials. Furthermore, activation is observed for the kinases p38α and PKA, which have been shown to play crucial roles in invasiveness and acquired Herceptin resistance (Donnelly et al., 2014; Gu et al., 2009). The most intense level of T-loop phosphorylation was observed for PKC, which is required for HER2-mediated NF-κB activation, thus mediating properties of malignancy, such as proliferation, invasiveness, and avoidance of apoptosis (Pan et al., 2016).

These results demonstrate the clinical potential of our technology to molecularly profile diseases such as breast cancer, being the most frequent cause of cancer deaths among women worldwide. It is a complex heterogeneous disease, comprising various subtypes with distinct genetic and morphological features, leading to differences in treatment response and clinical outcome. Consequently, present standard therapies are insufficient to treat metastatic disease and rationally chosen drug combinations are needed (Sachs et al., 2018; Zardavas et al., 2013). We argue that monitoring kinase activation in such a setting could have a profound impact, uncovering potential novel pharmaceutical targets.

DISCUSSION

In this study, we provide carefully optimized assays for T-loop phosphorylation on 178 protein kinases, accounting for roughly one-third of the human kinome. The strength of the technology lies in the combination of sensitivity, enabling substantial kinome coverage even from limited starting material (around 150–250 μg protein per sample) and throughput, using targeted SRM assays on a relatively simple triple-quadrupole MS instrument. Our approach is highly adaptable to various research questions and sample types as it provides enough sensitivity to reliably quantify kinase activation from limited sample amounts as encountered when working with primary cells or even clinical samples. The unbiased nature of the approach facilitates the detection of numerous understudied kinases (Table S8), alleviating the research bias that is still omnipresent in kinase research (Edwards et al., 2011). The use of sample fractionation or the enrichment for specific cell compartments such as nuclei or the cell membrane will further increase kinome coverage.

OPEN
ACCESS
CellPress

Applying this approach, we were able to detect 73 T-loop phosphorylations for 63 kinase groups reflecting 90 individual kinases across numerous cell types (primary and patient derived) (Table S5), which, to the best of our knowledge, presents the largest compendium of kinase activation sites reported to date. We demonstrate the utility of our technology for exclusive detection of activated kinases upon selected stimuli or specific treatment regimes. Our technology reveals, besides known signaling pathway activation, T-loop phosphorylations of the elusive RIPK1 S161, dynamic activation of primary blood platelets, rewiring of signal transduction in melanoma upon acquired drug resistance, and global kinase activity status in a breast cancer tumor biopsy sample. In conclusion, we present here a robust approach to quantify system-wide T-loop phosphorylations as a proxy for human protein kinase activity in various biological settings.

STAR★METHODS

Detailed methods are provided in the online version of this paper and include the following:

- KEY RESOURCES TABLE
- LEAD CONTACT AND MATERIALS AVAILABILITY
- EXPERIMENTAL MODEL AND SUBJECT DETAILS
  - Cell Models
  - Patients Samples
- METHOD DETAILS
  - TNF-α Stimulation and RIPK1 Inhibition
  - RIPK1 Immunoblot Analysis
  - PAR1 Activation of Pooled Platelets
  - Sample Preparation and Phosphopeptide Enrichment
  - LC-MS/MS Setup
  - Spectral Library Generation
  - SRM Assay Development
  - *T*-Loop Detection in Various Sample Types
  - Experimental Design and Replicates
  - SRM Data Assessment
  - Determining LOD and LOQ Values
  - Jurkat-Cells Shotgun Analysis
  - Web-Based Kinase Activity Prediction
- QUANTIFICATION AND STATISTICAL ANALYSIS
- DATA AND CODE AVAILABILITY

SUPPLEMENTAL INFORMATION

Supplemental Information can be found online at https://doi.org/10.1016/j.cels.2019.08.005.

ACKNOWLEDGMENTS

This work was supported by the Netherlands Organization for Scientific Research (NWO) through a VIDI grant for M.A. (723.012.102) and Proteins@-Work, a program of the National Roadmap Large-Scale Research Facilities of the Netherlands (project number 184.032.201). M.v.d.B. was supported by a fellowship from the Landsteiner Foundation for Blood Transfusion Research (LSBR 1517), K.K. and D.S.P. were supported by a grant from the Dutch Cancer Society (NKI-2013-5799) and a European Research Council grant under the European Union's Seventh Framework Programme (FP7/2007-2013)/ERC synergy grant agreement no. 319661 COMBATCANCER, and M.A. and D.S.P. were supported by a grant from the Dutch Cancer Society (10304). We would like to thank Dr. Wei Wu and T. Celine Mulder for providing cell pellets used in the proof of principle analysis and Lennart Mulder for preparation of the tumor biopsy sample.

AUTHOR CONTRIBUTIONS

Conceptualization, T.S., A.J.R.H., and M.A.; Methodology, T.S. and M.A.; Investigation, T.S., D.O.D., C.A.G.H.v.G., K.E.S., S.R., B.L.v.d.E., and K.K.; Writing – Original Draft, T.S. and M.A.; Writing – Review & Editing, T.S., E.H.L., M.v.d.B., D.S.P., A.J.R.H., and M.A.; Visualization, T.S. and D.O.D.; Funding Acquisition, M.v.d.B., D.S.P., A.J.R.H., and M.A.; Resources, E.H.L., M.v.d.B., and D.S.P.; Supervision, E.H.L., M.v.d.B., D.S.P., A.J.R.H., and M.A.

DECLARATION OF INTERESTS

Patent applicant, Utrecht Holding; name of inventor(s), M.A. and T.S.; application number, EP18179104.7; status of application, pending; specific aspect of the manuscript covered in the patent application, method for monitoring kinase activity in a sample. M.A. is a scientific advisor for Pepscope.

Received: October 16, 2018
Revised: June 13, 2019
Accepted: August 13, 2019
Published: September 11, 2019

REFERENCES

Abelin, J.G., Patel, J., Lu, X., Feeney, C.M., Fagbami, L., Creech, A.L., Hu, R., Lam, D., Davison, D., Pino, L., et al. (2016). Reduced-representation phosphosignatures measured by quantitative targeted ms capture cellular states and enable large-scale comparison of drug-induced phenotypes. Mol. Cell. Proteomics *15*, 1622–1641.

Annibaldi, A., and Meier, P. (2018). Checkpoints in TNF-induced cell death: implications in inflammation and cancer. Trends Mol. Med. *24*, 49–65.

Atkinson, B.T., Ellmeier, W., and Watson, S.P. (2003). Tec regulates platelet activation by GPVI in the absence of Btk. Blood *102*, 3592–3599.

Bantscheff, M., Eberhard, D., Abraham, Y., Bastuck, S., Boesche, M., Hobson, S., Mathieson, T., Perrin, J., Raida, M., Rau, C., et al. (2007). Quantitative chemical proteomics reveals mechanisms of action of clinical ABL kinase inhibitors. Nat. Biotechnol. *25*, 1035–1044.

Batth, T.S., Francavilla, C., and Olsen, J.V. (2014). Off-line high-pH reversed-phase fractionation for in-depth phosphoproteomics. J. Proteome Res. *13*, 6176–6186.

Blume-Jensen, P., and Hunter, T. (2001). Oncogenic kinase signalling. Nature *411*, 355–365.

Chang, C.Y., Picotti, P., Hüttenhain, R., Heinzelmann-Schwarz, V., Jovanovic, M., Aebersold, R., and Vitek, O. (2012). Protein significance analysis in selected reaction monitoring (SRM) measurements. Mol. Cell. Proteomics *11*, M111.014662.

Choi, M., Chang, C.-Y., Clough, T., Broudy, D., Killeen, T., MacLean, B., and Vitek, O. (2014). MSstats: an R package for statistical analysis of quantitative mass spectrometry-based proteomic experiments. Bioinformatics *30*, 2524–2526.

Collins, K.A.L., Stuhlmiller, T.J., Zawistowski, J.S., East, M.P., Pham, T.T., Hall, C.R., Goulet, D.R., Bevill, S.M., Angus, S.P., Velarde, S.H., et al. (2018). Proteomic analysis defines kinase taxonomies specific for subtypes of breast cancer. Oncotarget *9*, 15480–15497.

Cox, J., and Mann, M. (2008). MaxQuant enables high peptide identification rates, individualized p.p.b.-range mass accuracies and proteome-wide protein quantification. Nature Biotechnology *26*, 1367–1372.

Cristobal, A., Hennrich, M.L., Giansanti, P., Goerdayal, S.S., Heck, A.J., and Mohammed, S. (2012). In-house construction of a UHPLC system enabling the identification of over 4000 protein groups in a single analysis. Analyst *137*, 3541–3548.

de Graaf, E.L., Kaplon, J., Mohammed, S., Vereijken, L.A., Duarte, D.P., Redondo Gallego, L., Heck, A.J., Peeper, D.S., and Altelaar, A.F. (2015). Signal transduction reaction monitoring deciphers site-specific PI3K-mTOR/MAPK pathway dynamics in oncogene-induced senescence. J. Proteome Res. *14*, 2906–2914.

Degterev, A., Hitomi, J., Germscheid, M., Ch'en, I.L., Korkina, O., Teng, X., Abbott, D., Cuny, G.D., Yuan, C., Wagner, G., et al. (2008). Identification of RIP1 kinase as a specific cellular target of necrostatins. Nat. Chem. Biol. *4*, 313–321.

Desiere, F., Deutsch, E.W., King, N.L., Nesvizhskii, A.I., Mallick, P., Eng, J., Chen, S., Eddes, J., Loevenich, S.N., and Aebersold, R. (2006). The PeptideAtlas project. Nucleic Acids Res. *34*, D655–D658.

Ding, V.M., Boersema, P.J., Foong, L.Y., Preisinger, C., Koh, G., Natarajan, S., Lee, D.Y., Boekhorst, J., Snel, B., Lemeer, S., et al. (2011). Tyrosine phosphorylation profiling in FGF-2 stimulated human embryonic stem cells. PLoS One *6*, e17538.

Donnelly, S.M., Paplomata, E., Peake, B.M., Sanabria, E., Chen, Z., and Nahta, R. (2014). P38 MAPK contributes to resistance and invasiveness of HER2-overexpressing breast cancer. Curr. Med. Chem. *21*, 501–510.

Edwards, A.M., Isserlin, R., Bader, G.D., Frye, S.V., Willson, T.M., and Yu, F.H. (2011). Too many roads not taken. Nature *470*, 163–165.

Fabbro, D., Cowan-Jacob, S.W., and Moebitz, H. (2015). Ten things you should know about protein kinases: IUPHAR Review 14. Br. J. Pharmacol. *172*, 2675–2700.

Garay, J.P., and Gray, J.W. (2012). Omics and therapy - a basis for precision medicine. Mol. Oncol. *6*, 128–139.

Gross, S., Rahal, R., Stransky, N., Lengauer, C., and Hoeflich, K.P. (2015). Targeting cancer with kinase inhibitors. J. Clin. Invest. *125*, 1780–1789.

Grover, S.P., Bergmeier, W., and Mackman, N. (2018). Platelet signaling pathways and new inhibitors. Arterioscler. Thromb. Vasc. Biol. *38*, e28–e35.

Gu, L., Lau, S.K., Loera, S., Somlo, G., and Kane, S.E. (2009). Protein kinase A activation confers resistance to trastuzumab in human breast cancer cell lines. Clin. Cancer Res. *15*, 7196–7206.

Guo, T., Lee, S.S., Ng, W.H., Zhu, Y., Gan, C.S., Zhu, J., Wang, H., Huang, S., Sze, S.K., and Kon, O.L. (2011). Global molecular dysfunctions in gastric cancer revealed by an integrated analysis of the phosphoproteome and transcriptome. Cell. Mol. Life Sci. *68*, 1983–2002.

Holler, N., Zaru, R., Micheau, O., Thome, M., Attinger, A., Valitutti, S., Bodmer, J.L., Schneider, P., Seed, B., and Tschopp, J. (2000). Fas triggers an alternative, caspase-8-independent cell death pathway using the kinase RIP as effector molecule. Nat. Immunol. *1*, 489–495.

Käll, L., Canterbury, J.D., Weston, J., Noble, W.S., and MacCoss, M.J. (2007). Semi-supervised learning for peptide identification from shotgun proteomics datasets. Nat. Methods *4*, 923–925.

Kemper, K., Krijgsman, O., Cornelissen-Steijger, P., Shahrabi, A., Weeber, F., Song, J.Y., Kuilman, T., Vis, D.J., Wessels, L.F., Voest, E.E., et al. (2015). Intra- and inter-tumor heterogeneity in a vemurafenib-resistant melanoma patient and derived xenografts. EMBO Mol. Med. *7*, 1104–1118.

Kostenko, S., Dumitriu, G., and Moens, U. (2012). Tumour promoting and suppressing roles of the atypical MAP kinase signalling pathway ERK3/4-MK5. J. Mol. Signal. *7*, 9.

Lachmann, A., and Ma'ayan, A. (2009). KEA: kinase enrichment analysis. Bioinformatics *25*, 684–686.

Lahiry, P., Torkamani, A., Schork, N.J., and Hegele, R.A. (2010). Kinase mutations in human disease: interpreting genotype-phenotype relationships. Nat. Rev. Genet. *11*, 60–74.

Maclean, B., Tomazela, D.M., Abbatiello, S.E., Zhang, S., Whiteaker, J.R., Paulovich, A.G., Carr, S.A., and Maccoss, M.J. (2010a). Effect of collision energy optimization on the measurement of peptides by selected reaction monitoring (SRM) mass spectrometry. Anal. Chem. *82*, 10116–10124.

MacLean, B., Tomazela, D.M., Shulman, N., Chambers, M., Finney, G.L., Frewen, B., Kern, R., Tabb, D.L., Liebler, D.C., and MacCoss, M.J. (2010b). Skyline: an open source document editor for creating and analyzing targeted proteomics experiments. Bioinformatics *26*, 966–968.

Manning, G., Whyte, D.B., Martinez, R., Hunter, T., and Sudarsanam, S. (2002). The protein kinase complement of the human genome. Science *298*, 1912–1934.

Miller, M.L., Jensen, L.J., Diella, F., Jørgensen, C., Tinti, M., Li, L., Hsiung, M., Parker, S.A., Bordeaux, J., Sicheritz-Ponten, T., et al. (2008). Linear motif atlas for phosphorylation-dependent signaling. Sci. Signal. *1*, ra2.

Nesvizhskii, A.I., and Aebersold, R. (2005). Interpretation of shotgun proteomic data: the protein inference problem. Mol. Cell. Proteomics *4*, 1419–1440.

Nolen, B., Taylor, S., and Ghosh, G. (2004). Regulation of protein kinases; controlling activity through activation segment conformation. Mol. Cell *15*, 661–675.

Pan, D., Zhu, Y., Zhou, Z., Wang, T., You, H., Jiang, C., and Lin, X. (2016). The CBM complex underwrites NF-κB activation to promote HER2-associated tumor malignancy. Mol. Cancer Res. *14*, 93–102.

Patricelli, M.P., Nomanbhoy, T.K., Wu, J., Brown, H., Zhou, D., Zhang, J., Jagannathan, S., Aban, A., Okerberg, E., Herring, C., et al. (2011). In situ kinase profiling reveals functionally relevant properties of native kinases. Chem. Biol. *18*, 699–710.

Post, H., Penning, R., Fitzpatrick, M.A., Garrigues, L.B., Wu, W., MacGillavry, H.D., Hoogenraad, C.C., Heck, A.J., and Altelaar, A.F. (2017). Robust, sensitive, and automated phosphopeptide enrichment optimized for low sample amounts applied to primary hippocampal neurons. J. Proteome Res. *16*, 728–737.

Ruprecht, B., Zecha, J., Heinzlmeir, S., Médard, G., Lemeer, S., and Kuster, B. (2015). Evaluation of kinase activity profiling using chemical proteomics. ACS Chem. Biol. *10*, 2743–2752.

Sachs, N., de Ligt, J., Kopper, O., Gogola, E., Bounova, G., Weeber, F., Balgobind, A.V., Wind, K., Gracanin, A., Begthel, H., et al. (2018). A living biobank of breast cancer organoids captures disease heterogeneity. Cell *172*, 373–386.e10.

Schmidlin, T., Garrigues, L., Lane, C.S., Mulder, T.C., van Doorn, S., Post, H., de Graaf, E.L., Lemeer, S., Heck, A.J., and Altelaar, A.F. (2016). Assessment of SRM, MRM(3), and DIA for the targeted analysis of phosphorylation dynamics in non-small cell lung cancer. Proteomics *16*, 2193–2205.

Sharifnia, T., Rusu, V., Piccioni, F., Bagul, M., Imielinski, M., Cherniack, A.D., Pedamallu, C.S., Wong, B., Wilson, F.H., Garraway, L.A., et al. (2014). Genetic modifiers of EGFR dependence in non-small cell lung cancer. Proc. Natl. Acad. Sci. USA *111*, 18661–18666.

Shrivastava, A., and Gupta, V.B. (2011). Methods for the determination of limit of detection and limit of quantitation of the analytical methods. Chronicles Young Sci. *2*, 21–25.

Soste, M., Hrabakova, R., Wanka, S., Melnik, A., Boersema, P., Maiolica, A., Wernas, T., Tognetti, M., von Mering, C., and Picotti, P. (2014). A sentinel protein assay for simultaneously quantifying cellular processes. Nat. Methods *11*, 1045–1048.

Stuhlmiller, T.J., Miller, S.M., Zawistowski, J.S., Nakamura, K., Beltran, A.S., Duncan, J.S., Angus, S.P., Collins, K.A., Granger, D.A., Reuther, R.A., et al. (2015). Inhibition of Lapatinib-induced kinome reprogramming in ERBB2-positive breast cancer by targeting BET family bromodomains. Cell Rep. *11*, 390–404.

Stuhlmiller, T.J., Zawistowski, J.S., Chen, X., Sciaky, N., Angus, S.P., Hicks, S.T., Parry, T.L., Huang, W., Beak, J.Y., Willis, M.S., et al. (2017). Kinome and transcriptome profiling reveal broad and distinct activities of erlotinib, sunitinib, and sorafenib in the mouse heart and suggest cardiotoxicity From combined signal transducer and activator of transcription and epidermal growth factor receptor inhibition. J. Am. Heart Assoc. *6*.

van den Eshof, B.L., Hoogendijk, A.J., Simpson, P.J., van Alphen, F.P.J., Zanivan, S., Mertens, K., Meijer, A.B., and van den Biggelaar, M. (2017). Paradigm of biased PAR1 (protease-activated receptor-1) activation and inhibition in endothelial cells dissected by phosphoproteomics. Arterioscler. Thromb. Vasc. Biol. *37*, 1891–1902.

van Ramshorst, M.S., van Werkhoven, E., Honkoop, A.H., Dezentjé, V.O., Oving, I.M., Mandjes, I.A., Kemper, I., Smorenburg, C.H., Stouthard, J.M., Linn, S.C., et al. (2016). Toxicity of dual HER2-blockade with pertuzumab added to

anthracycline versus non-anthracycline containing chemotherapy as neoadjuvant treatment in HER2-positive breast cancer: the TRAIN-2 study. Breast *29*, 153–159.

Wagle, N., Emery, C., Berger, M.F., Davis, M.J., Sawyer, A., Pochanard, P., Kehoe, S.M., Johannessen, C.M., Macconaill, L.E., Hahn, W.C., et al. (2011). Dissecting therapeutic resistance to RAF inhibition in melanoma by tumor genomic profiling. J. Clin. Oncol. *29*, 3085–3096.

Wagner, E.F., and Nebreda, A.R. (2009). Signal integration by JNK and p38 MAPK pathways in cancer development. Nat. Rev. Cancer *9*, 537–549.

Xiao, Y., Guo, L., and Wang, Y. (2014). A targeted quantitative proteomics strategy for global kinome profiling of cancer cells and tissues. Mol. Cell. Proteomics *13*, 1065–1075.

Zagorac, I., Fernandez-Gaitero, S., Penning, R., Post, H., Bueno, M.J., Mouron, S., Manso, L., Morente, M.M., Alonso, S., Serra, V., et al. (2018). In vivo phosphoproteomics reveals kinase activity profiles that predict treatment outcome in triple-negative breast cancer. Nat. Commun. *9*, 3501.

Zardavas, D., Baselga, J., and Piccart, M. (2013). Emerging targeted agents in metastatic breast cancer. Nat. Rev. Clin. Oncol. *10*, 191–210.

STAR★METHODS

KEY RESOURCES TABLE

REAGENT or RESOURCE	SOURCE	IDENTIFIER
Antibodies		
Purified Mouse Anti-RIP	BD Biosciences	Cat#610458; RRID: AB_397831
GAPDH antibody [GT239] (mouse)	GeneTex	Cat# GTX627408, RRID: AB_11174761
Goat Anti-Mouse Immunoglobulins/HRP	Agilent Technologies	P044701-2
Biological Samples		
Patient derived platelet cells	Sanquin Blood Bank	https://www.sanquin.org/working-at/production/blood-bank
Patient derived biopsy from primary breast tumors	TRAIN2 clinical trial	https://clinicaltrials.gov/ct2/show/NCT01996267
Chemicals, Peptides, and Recombinant Proteins		
SpikeMix™ Kinase Activation Loops (Human) - heavy	JPT Germany	SPT-KAL-POOL-L-100pm
HRM Calibration Kit	Biognosys	Ki-3003
TNF-α	PeproTech	300-01A
PLX-4720	Selleckchem	S1152
PAR1 mimicking hexapeptide SFLLRN-NH2	Peptides International	PAR-3676-PI
Nec-1	Merck	Cat#480065
Tirofiban	Iroko cardio, UK	CAS 0144494-65-5
Critical Commercial Assays		
Caspase-3/7 green apoptosis reagent	Essen Bioscience	Cat#4440
Sep-Pak C18 1 cc Vac Cartridge	Waters	WAT023590
Bio-Rad Protein Assay Kit I	Bio-Rad	5000001
PhosSTOP™	Merck	4906837001
cOmplete™, Mini, EDTA-free Protease Inhibitor Cocktail	Merck	11836170001
AssayMap Cartridge Rack, Fe(III)-NTA 5 μL	Agilent Technologies	Cat#G5496-60085
Pierce™ ECL Plus Western Blotting Substrate	Thermo Scientific™	32132
Deposited Data		
.raw and .wiff data of all experiments	SRMAtlas	http://www.peptideatlas.org/PASS/PASS01234
Analyzed SRM data within Skyline daily framework	SRMAtlas	http://www.peptideatlas.org/PASS/PASS01234
MaxQuant Database search output performed on fractionated Jurkat lysates for motiv analysis	SRMAtlas	http://www.peptideatlas.org/PASS/PASS01234
NetworKIN kinase activity prediction	This paper	Table S6
Quantitative and statistical analysis of SRM assays performed in MSstats	This Paper	Table S5
KEA2 kinase activity predicton	This Paper	Table S6
Experimental Models: Cell Lines		
Jurkat	DSMZ	ACC 282
HEK293T	ATCC	Cat#ATCC CRL-3216
PC9	Sigma-Aldrich	90071810-1 VL
M026	Laboratory of Daniel Peeper NKI	https://www.nki.nl/divisions/molecular-oncology-immunology/peeper-d-group/
Software and Algorithms		
Skyline daily	MacLean et al., 2010b	https://skyline.ms/project/home/software/Skyline/begin.view
MaxQuant (version 1.6.1.0)	Cox and Mann, 2008	https://www.maxquant.org/
MSStats	Choi et al., 2014	http://msstats.org/

(*Continued on next page*)

Continued		
REAGENT or RESOURCE	SOURCE	IDENTIFIER
MASCOT	Matrix Science	http://www.matrixscience.com/
Proteome Discoverer	Thermo Fisher	www.thermofisher.com
NetworKin	Miller et al., 2008	http://netphorest.info
KEA2	Lachmann, and Ma'ayan, 2009	http://www.maayanlab.net/KEA2
Other		
25 cm, 75 μm ID PepMap RLSC, C18, 100 Å, 2 μm particle size column	Thermo Scientific	ES802

LEAD CONTACT AND MATERIALS AVAILABILITY

Further information and requests for resources and reagents should be directed to and will be fulfilled by the Lead Contact, Maarten Altelaar (m.altelaar@uu.nl).

EXPERIMENTAL MODEL AND SUBJECT DETAILS

Cell Models

PC9 NSCLC cells (originating from human adenocarcinoma, male) were purchased from Sigma-Aldrich (cat# 90071810-1 VL) and were cultured in standard Roswell Park Memorial Institute medium 1640 medium (Lonza), containing 10% FBS (Thermo), 2 mM L-glutamine and 1% penicillin/streptomycin (Lonza), at 37°C in a humidified atmosphere containing 5% CO_2. Cells were detached from the culture surface using trypsin (Lonza), and washed three times with PBS before lysis.

Hek293T cells (embryonic kidney of female fetus purchased from ATCC, cat# ATCC CRL-3216) were seeded at 15% density in 15-cm plates, allowed to adhere in full DMEM (Lonza) containing 10% heat-inactivated fetal bovine serum (Gibco), 2 mM L-glutamine (Lonza) and 20 mM HEPES (Sigma-Aldrich), and cultured to ~90% confluence over 2.5 days. Twelve hours prior to harvesting, growth medium was replaced with fresh pre-warmed full DMEM. At harvesting, no dead or floating cells were visible by microscopic examination. Cells were washed twice with ice-cold PBS on-plate, detached by trypsin (Lonza), and collected by low-speed centrifugation at 200g for 5 min.

Jurkat cells (male, purchased from DSMZ, ACC 282) were cultured in RPMI-1640 media complemented with l-glutamine, 10% fetal bovine serum, and PenStrep at a density between $5x10^5$ and $5x10^6$ per ml.

PDX melanoma cell lines (M026, M026R) were cultured in RPMI 1640 with HEPES and L-Glutamine supplemented with 10% fetal bovine serum and Penicillin-Streptomycin. The resistant cell lines were grown in presence of 1 μM PLX-4720 (Selleckchem). When specified, the cell lines were treated with an IC_{50} dose (1 μM) of the inhibitors PLX-4720. All cell pellets were stored at -80°C prior to cell lysis.

Pooled platelet concentrates in plasma (platelet concentrates from 5 donors in ABO blood-group-matched plasma) were obtained from the Dutch Bloodbank (Sanquin). Multiple platelet concentrates were pooled in three separate pools and processed independently. Platelets were isolated as described before (van den Eshof et al., 2017). Briefly, platelet concentrates were centrifuged for 20 min at 120g, to remove remaining red and white blood cells. Next, platelets were washed twice with isolation buffer (36 mM citric acid, 103 mM NaCl, 5 mM KCl, 5 mM ethylenediaminetetraacetic acid (EDTA), 5.6 mM D-glucose, pH 6.5, containing 0.35% [w/v] bovine serum albumin (BSA)) by centrifugation for 10 min at 2,000g. Platelets were washed once with Tyrode's Solution (Sigma), centrifuged for 10 min at 2,000g and resuspended to a final concentration of 2×10^8 platelets/mL in Tyrode's Solution supplemented with tirofiban (1:1000) (Iroko cardio, UK).

Patients Samples

Frozen biopsies were obtained from primary breast tumors of patients undergoing neoadjuvant chemotherapy and dual HER2 blockade in the TRAIN2 trial (NCT01996267) (van Ramshorst et al., 2016). The study had been approved by the ethical committee and informed consent was obtained from all patients. Approximately fifteen 30 μm frozen sections were prepared with a cryostat microtome for protein extraction. R/DNase free H_2O was used as adhesive instead of a polymer adhesive to eliminate any traces of polymers in the protein extract. About halfway through the biopsy one or two sections of 6-8 μm were prepared for hematoxylin and eosin (HE) staining. The HE stained sections were reviewed by a pathologist to estimate the tumor cell percentage of the tissue.

METHOD DETAILS

TNF-α Stimulation and RIPK1 Inhibition

One day prior stimulation, Jurkat cells were seeded at a concentration of $1x10^6$ per ml in fresh media. TNF-α (PeproTech, USA) was added at a concentration of 100 U/ml for 8 h, after which the cells were washed with ice cold PBS and snap frozen. For the apoptosis

assay the Jurkat cells were plated at a density of $3x10^4$ cells in a 96 well plate coated with 0.01% poly-l-ornithine. Cells were pre-incubated with 0.5 μM Necrostatin-1 (Merck), and then stimulated with TNF-α at a concentration of 100U/ml in the presence of 2.5 μM Caspase-3/7 green apoptosis reagent (Essen Bioscience). Apoptosis was monitored using the IncuCyte Zoom system (Essen Bioscience) every hour for 48 h. Each well was divided into four views, and the number of green fluorescent cells was counted using the IncuCyte Zoom software (Essen Bioscience).

RIPK1 Immunoblot Analysis

Cell pellets were lysed by sonication in Urea buffer (8 M Urea, 50 mM ammonium bicarbonate, protease inhibitor Cocktail Complete (Roche), phosphatase inhibitors PhosSTOP (Roche)) and protein concentration was determined using a Bradford assay (Bio-Rad). Immunoblotting was performed using 12% Criterion XT precast gel (Bio-Rad) and nitrocellulose membrane. All blocking steps were performed using 5% milk in TBS-Tween (Tris-buffered saline (pH 7.5) with 0.1% Tween (Biorad)). GAPDH (GeneTex, RRID: AB_11174761) and RIPK1 (BD Biosciences, RRID: AB_397831) were used as primary antibodies, goat anti-mouse immunoglobulins/HRP (Agilent Technologies) was used as secondary antibody. Protein detection was performed using the ECL Plus Substrate (Pierce) and an Amersham Imager AI600 for chemiluminescence imaging.

PAR1 Activation of Pooled Platelets

Platelets were activated with 50 μM PAR1 peptide (SFLLRN-NH2, Peptides international) or negative control (Tyrode's solution) for 2 or 5 min at 37°C in a thermomixer (Eppendorf). Activation was stopped by lysis of the platelets by addition of 2 times (% v/v) 1.5x SDC lysis buffer (1.5% SDC, 15 mM TCEP, 60 mM Chloroactamide, 150 mM tris(hydroxymethyl)aminomethane (Tris) pH 8.5, 1.5% phosphatase and protease inhibitor cocktail (Thermo Scientific)). Platelet lysates were snap frozen and stored at -80°C.

Sample Preparation and Phosphopeptide Enrichment

Frozen cell pellets and patient biopsy were lysed, reduced and alkylated in lysis buffer (1% sodium deoxycholate (SDC), 10 mM tris(2-carboxyethyl)phosphinehydrochloride (TCEP)), 40 mM chloroacetamide (CAA), and 100 mM TRIS, pH 8.0 supplemented with phosphotase inhibitor (PhosSTOP, Roche) and protease inhibitor (cOmplete mini EDTA-free, Roche). Cells were heated at 95°C and sonicated with a Bioruptor Plus (Diagenode) for 15 cycles of 30 s. Bradford protein assay (Bio-Rad Protein Assay Kit I, Bio-Rad) was used to determine protein amount. To avoid digestion bias, samples were split into aliquots containing equal amount of protein. Proteins were digested overnight at 37°C with trypsin (Sigma-Aldrich) with an enzyme/substrate ratio of 1:50 and lysyl endopeptidase (Wako) with an enzyme/substrate ratio of 1:75. SDC was precipitated with 2% formic acid (FA) and samples were desalted using Sep-Pak C18 cartridges (Waters). Subsequently samples were dried in vacuo and stored at -80°C until further use.

Phosphorylated peptides were enriched using Fe(III)-NTA cartridges 5 μL (Agilent technologies) in an automated fashion using the AssayMAP Bravo Platform (Agilent Technologies) as previously described (Post et al., 2017). For all samples, 250 μg of peptides were used as input for one cartridge, except for the biopsy sample where only approximately 150 μg of protein was available per cartride in order to allow for analyses in both, survey and quantification mode. In brief, Fe(III)-NTA cartridges were primed with 200 μL of 0.1% TFA in ACN and equilibrated with 250 μL of loading buffer (80% ACN/0.1% TFA). Samples were dissolved in 200 μL of loading buffer containing 100 fmol synthetic isotope labelled t-loop standard peptides and loaded onto the cartridge at a loading speed of 5 μL/min. Subsequently columns were washed with 250 μL loading buffer and eluted with 35 μL of 10% ammonia directly into 35 μL of 10% formic acid. Samples were dried down and stored at −80°C until LC–MS analysis.

LC-MS/MS Setup

Spectral libraries were partly acquired on a TripleTOF 5600 (Sciex) coupled to an Agilent 1290 Infinity System (Agilent Technologies) adapted to nanoflow conditions by using a split flow setup as described in Cristobal et al. (2012). The system was operated with in-house packed trap column (Dr. Maisch Reprosil C18, 3 μm, 2 cm × 100 μm) and analytical column (Agilent Poroshell 120 EC-C18, 2.7 μm, 50 cm × 75 μm). The split flow was adapted to achieve 300 nl/min flow at the front end of the column upon applying a flow rate of 0.2 mL/min. 0.6% acetic acid in water (Milli-Q, Millipore) was used as buffer A an 0.6% acetic acid, 80% ACN was used as buffer B. Upon injection, peptides were trapped at 5 μL/min during 5 min with 100% solvent A (0.1% FA in water) before being separated on the analytical column.

All remaining measurements were executed on a TSQ-Vantage (Thermo Fisher) coupled to an Easy-nLC 1000 (Proxeon, Odense, DK). LC configuration was in one-column setup (25 cm, 75 μm ID PepMap RLSC, C18, 100 Å, 2 μm particle size column (Thermo Scientific, Odense, DK)). Formic acid (0.1%, Merck, Darmstadt, Germany) in deionized water (Biosolve, Valkenswaard, NL, ULC/MS grade) was used as solvent A, 0.1% formic acid in acetonitrile (Biosolve, Valkenswaard, NL, ULC/MS grade) as solvent B. All measurements were performed at 200 nl/min flow rate and all samples were analyzed with injection volumes of 2 μL containing 10% FA.

Spectral Library Generation

SRM assay development was guided by a spectral library generated in house. This provided confirmation of the synthesized peptide sequence and essential information about LC and MS characteristics of each peptide. For this spectral library generation, the heavy labeled phosphopeptides were mixed with 1x HRM Calibration KIT and analyzed in data-dependent mode on two different LC-MS setups. (1) Crude peptides were analyzed on a 5600 TripleTOF (Sciex). LC-MS setup and data acquisition methods were used as

previously described (Schmidlin et al., 2016). In brief, peptides were separated on a 2 h gradient analyzed in TOP20 mode (selection criteria: intensity > 50 cps, charge state ≥ 2+, dynamic exclusion 15 s). (2) Crude peptides were analyzed on a TSQ Vantage (Thermo) in data-dependent acquisition mode operated at TOP2. Survey scans were acquired in Q3MS mode (1.5 s, 0.4 Da fwhm) spanning the 375-1350m/z range. The TOP2 most abundant ions were analyzed in MS/MS mode (selection criteria: intensity > 1000 counts, 5 repeats at 3 s, 2 min dynamic exclusion). Results of both acquisition types were subjected to database search using Mascot accessed by Proteome Discoverer (version 1.4). Parameters were set to tryptic digest, allowing for up to three missed cleavages, using carbamidomethyl cysteine as fixed modification and allowing for serine/threonine/tyrosine phosphorylation methionine oxidation and C-terminal isotope labels. Precursor mass tolerance and MS/MS tolerance were set to 50 ppm and 0.15 Da respectively for TripleTOF files and to 0.9 Da for TSQ files. Results were filtered using Percolator (Käll et al., 2007) to an FDR below 1%. Spectral libraries were built in Skyline from both searches.

SRM Assay Development

All assays were developed and optimized on a TSQ Vantage as previously described (de Graaf et al., 2015; Soste et al., 2014). In brief, the most intense fragment ions found in the spectral libraries were directly used as initial transitions, multiplexing up to 3-10 transitions per precursor. Those initial SRM assays were applied to the synthetic peptide library enabling subsequent optimization of multiple parameters such as collision energy and RT scheduling. Initial assays for a few peptides not identified in either of the spectral library were constructed from theoretically possible y- and b-ions in combination with their most likely precursor charge states. Extensive manual validation of phosphosites localization isomers was performed, including the use of site determining ions as transitions. Collision energies were optimized for each transition individually in an empirical way assisted by Skyline (Maclean et al., 2010a). Instrument specific CE parameters were used as a starting point (CE = 0.03m/z + 2.905 for doubly charged precursors and CE = 0.038m/z + 2.281 for precursor charges of three and higher) to scan through different normalized collision energy values using a step size of one.

T-Loop Detection in Various Sample Types

Dried samples were reconstituted in 3 μL of 10% formic acid, containing 0.1x/μL iRT peptides. Injection volumes for all analyses were kept at 2 μL. LC-MS analysis contained the following steps: Analytical column equilibration (3 μL 100% Buffer A at 600 bars) followed by sample loading onto the column (loading volume 6 μL at 600 bars). Phosphopeptides were separated on a gradient from 2% to 25% B in 100 min, followed by a column washing step ramping up from 25% to 100% B in 5 min followed by 100% B for 15 min. To avoid carryover and monitor LC performance at least 1 BSA run was scheduled after each analysis. Samples within one experiment were analyzed in randomized order by defining and injection order based on random numbers prior to the analysis. Retention time scheduling was dynamically adapted to reflect the instrument state prior to sample analysis. Low confidence survey runs applied low transition numbers per peptide (~3) in short scheduling windows (4 min) applying long cycle times (4 s). High confidence quantification runs applied less target peptides with a higher number of transitions per peptide (up to 7, including phosphosite localization specific ions), longer scheduling windows (6-10 min) and shorter cycle times (2.5-3 s).

Experimental Design and Replicates

Proof of concept experiments for Jurkat, PC9 and Hek293T cells were based on a single cell culture experiment (n = 1) as they did not involve accurate quantification. Sample preparation was performed in at least 2 replicas to facilitate separation into survey and quantification runs. Quantitative experiments were performed in triplicates (n = 3) on a cell culture level including lysis and tryptic digestion. For each sample, two processing replicas were prepared on the level of phosphopeptide enrichment which were independently analyzed by LC-MS resulting in n = 6 injection replicas per condition. Additional phosphopeptide enrichments were performed for survey analyses when sufficient sample amount was available. To avoid missing condition-specific kinase activation states at least one sample per condition was measured in survey mode. Specific experimental designs were as follows: for TNF-α stimulation independent cell culture of n = 3 with TNF-α stimulation and n = 3 without any stimulation were performed. Likewise, PDX-derived cells were grown independently in triplicates (n = 3) for each of the 3 conditions respectively (n = 9). Analysis of the breast cancer needle biopsy was performed from a single sample (n = 1). The sample was split into 2 processing replicates prior to phosphopeptide enrichment to allow for analysis in survey and quantification mode.

SRM Data Assessment

All SRM experiments were analyzed using Skyline (MacLean et al., 2010b). Signal quality was assessed visually, primarily relying on sequence similarity between heavy labeled standard peptides and endogenous peptides. Key points used were perfect co-elution of both peptide forms in terms of retention time and peak shape in combination with a high similarity of the relative intensities of transitions found in the heavy and the endogenous peptides (rdotp > 0.9). Signals for endogenous peptide forms not matching these criteria were excluded, unless the result indicated a clear on/off state between different conditions (RIPK1 and ERK4) in which case noise levels were used as a quantitative readout.

Determining LOD and LOQ Values

LOD and LOQ values of selected peptides were determined using dilution series of stable isotope labeled peptides spiked into Jurkat matrix samples. Jurkat cells were cultured, lysed and digested as described above. 200 μg of cell digest were enriched for

phosphopeptide by Fe(III)-NTA and spiked with increasing amounts of the heavy isotope labeled phosphopeptide library (10 amol, 100 amol, 1 fmol, 5 fmol, 10, fmol, 50 fmol, 100 fmol). Samples for each quantity of heavy peptide spike-ins were prepared in triplicates. A subset of 44 heavy isotope labeled peptides were quantified in all samples by scheduled SRM using 3-7 transitions per peptide. Summed areas under the curve of all transitions were used as quantitative readout as reported by Skyline. Peaks were assessed visually, and representative noise area values were selected for spike-in amounts below the LOD. Linear regressions were performed for each peptide individually per sample replicate as well as combined for all replicas. LOD and LOQ values were determined according to the following equations: $LOD = 3S_a/b$ and $LOQ = 10S_a/b$ using b as the sensitivity (slope of the linear regression of all replicas combined) and S_a as the standard deviation of the intercept (determined for each regression individually) (Shrivastava and Gupta, 2011). To avoid skewing of the linear regressions, for each peptide only the highest spike-in amount below the LOD was included in the analysis.

Jurkat-Cells Shotgun Analysis

2 mg of cell digest from TNF-α treated Jurkat cell lines was fractionated on a high-pH (HpH) reversed-phase C18 column (Gemini 3 μm C18 110 Å, 100 × 1.0 mm, Phenomenex) coupled to an Agilent 1100 series (Agilent Technologies) on a 60 min gradient. 67 fractions of 1 min each were collected and concatenated into five pools as previously described (Batth et al., 2014). These were dried down in vacuo and subjected to phosphopeptide enrichment as described above. DDA analysis was performed on a Q Exactive HF (Thermo Scientific) coupled to an Easy-nLC 1000 (Proxeon, Odense, DK), configured as described above. All LC settings and methods including analysis time and gradient were identical to the ones used during the SRM analysis. The mass spectrometer was operated in data-dependent acquisition mode containing a survey scan from 375 to 1600m/z (resolution 60,000, max injection time 20 ms, AGC target 3e6) acquired in profile mode. MS/MS spectra (HCD, 27% normalized collision energy at a target value of 50,000 ions, resolution 30,000) were recorded for the 12 most intense peaks using a 1.4m/z isolation window. Data was analyzed by Maxquant (version 1.6.1.0) with the integrated Andromeda search engine. Trypsin was specified as enzyme and up to two missed cleavages were allowed. Cysteine carbamidomethylation was set as a fixed modification, while methionine oxidation and protein N-term acetylation were set as variable modifications. Phosphorylation on serine, threonine and tyrosine was also selected as variable modification. The mass tolerance was set to 4.5 ppm for precursor ions, and to 20 ppm (FTMS) for fragment ions. Peptide and protein identification were set to 1% FDR, and the minimum score for modified peptides was set to 40.

Web-Based Kinase Activity Prediction

KEA2 (http://www.maayanlab.net/KEA2) was used to predict kinase activity from shotgun data using enrichment analysis (Lachmann and Ma'ayan, 2009). Phosphosites detected by MaxQuant were used as data input. Enrichment analysis was performed using *Literature Based Kinase-Substrate Library with Phosphosites* option. NetworKIN analysis was performed on the same dataset (http://netphorest.info) (Miller et al., 2008). In the subsequent data visualization, only the highest score detected per kinase was considered.

QUANTIFICATION AND STATISTICAL ANALYSIS

For all SRM assays, the ratios between analyte and internal standard were used as quantitative readout. Unless otherwise specified significance analysis was performed with MSstats (Chang et al., 2012). The analysis entailed $\log_2$-transformation of the intensity values, followed by testing for abundance differences between different conditions using a linear-mixed effects model. An arbitrary cutoff of $p \leq 0.05$ was considered significant. For individual bar plots quantitative peptide abundance were exported from Skyline (normalized based on internal standards). Bars represent average and error bars represent standard deviation. Significance is indicated according to the adjusted p-value output provided by MSstats.

DATA AND CODE AVAILABILITY

The datasets generated during this study are available at Peptide Atlas PASS01234 (http://www.peptideatlas.org/PASS/PASS01234).

Cell Systems, Volume *9*

Supplemental Information

High-Throughput Assessment
of Kinome-wide Activation States

Thierry Schmidlin, Donna O. Debets, Charlotte A.G.H. van Gelder, Kelly E. Stecker, Stamatia Rontogianni, Bart L. van den Eshof, Kristel Kemper, Esther H. Lips, Maartje van den Biggelaar, Daniel S. Peeper, Albert J.R. Heck, and Maarten Altelaar

- Supplementary Information –

High-throughput assessment of kinome-wide activation states

Thierry Schmidlin[1,2], Donna O. Debets[1,2], Charlotte A.G.H. van Gelder[1,2], Kelly E. Stecker[1,2], Stamatia Rontogianni[1,2], Bart L. van den Eshof[3], Kristel Kemper[4], Esther H. Lips[5], Maartje van den Biggelaar[3], Daniel S. Peeper[4], Albert J.R. Heck[1,2] and Maarten Altelaar[1,2,6,#,*]

1 Biomolecular Mass Spectrometry and Proteomics, Bijvoet Center for Biomolecular Research and Utrecht Institute for Pharmaceutical Sciences, Utrecht University, Padualaan 8, 3584 CH Utrecht, The Netherlands

2 Netherlands Proteomics Center, Padualaan 8, 3584 CH Utrecht, The Netherlands

3 Sanquin Research, Department of Molecular and Cellular Hemostasis, Amsterdam, the Netherlands.

4 Division of Molecular Oncology, Oncode Institute, The Netherlands Cancer Institute, 1066 CX Amsterdam, the Netherlands

5 Department of Molecular Pathology, The Netherlands Cancer Institute, 1066 CX Amsterdam, the Netherlands.

6 Mass Spectrometry and Proteomics Facility, The Netherlands Cancer Institute, 1066 CX Amsterdam, the Netherlands

[#] To whom correspondence should be addressed: m.altelaar@uu.nl

* Lead contact

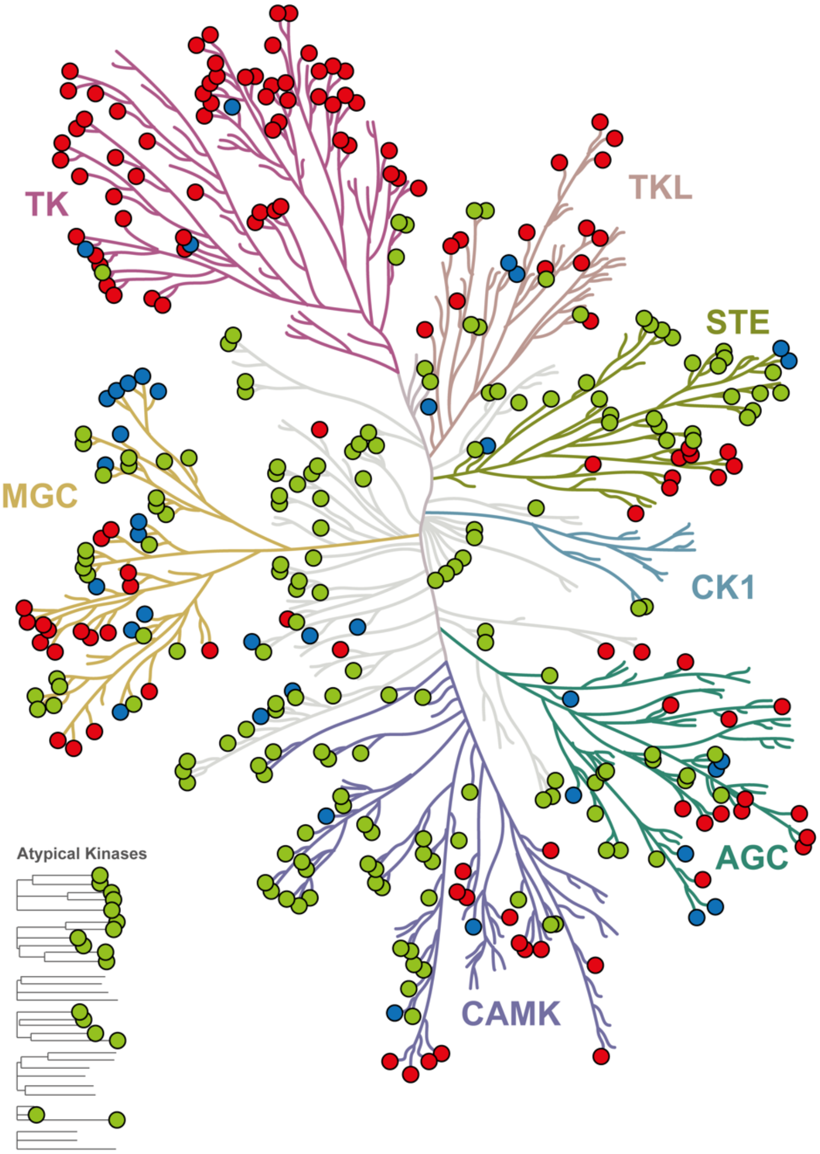

Figure S1, Related to Figure 1: Depiction of the human kinome indicating all kinases currently included in the t-loop screening method (blue circle), kinases being classified as understudied by Collins et al. (green circle) and the overlap of the two (red circles).

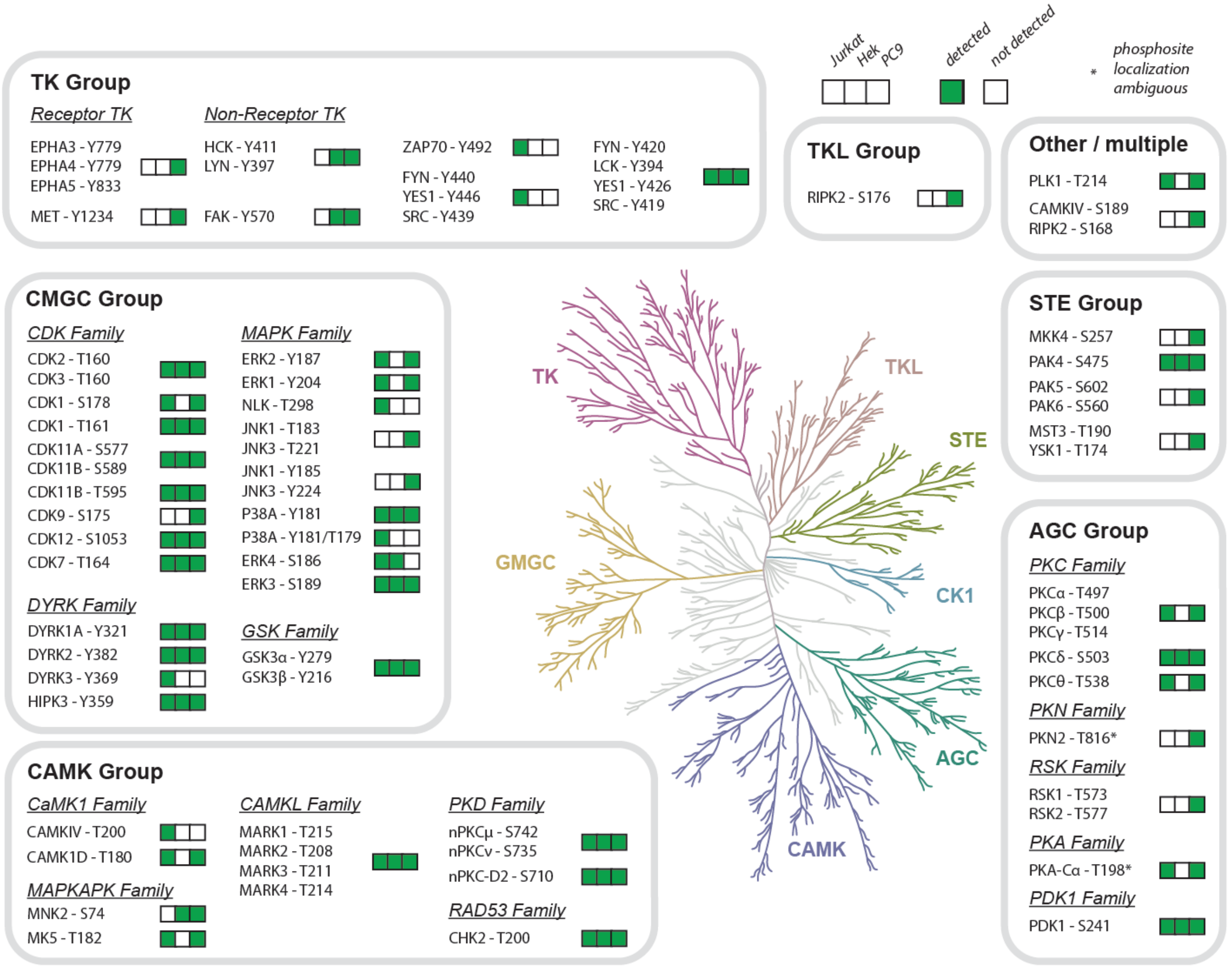

Figure S2, Related to Figure 1: Compendium of t-loop phosphorylations detected in Jurkat, HEK293T and PC9 cell lines cultured under standard conditions without any form of stimulation.

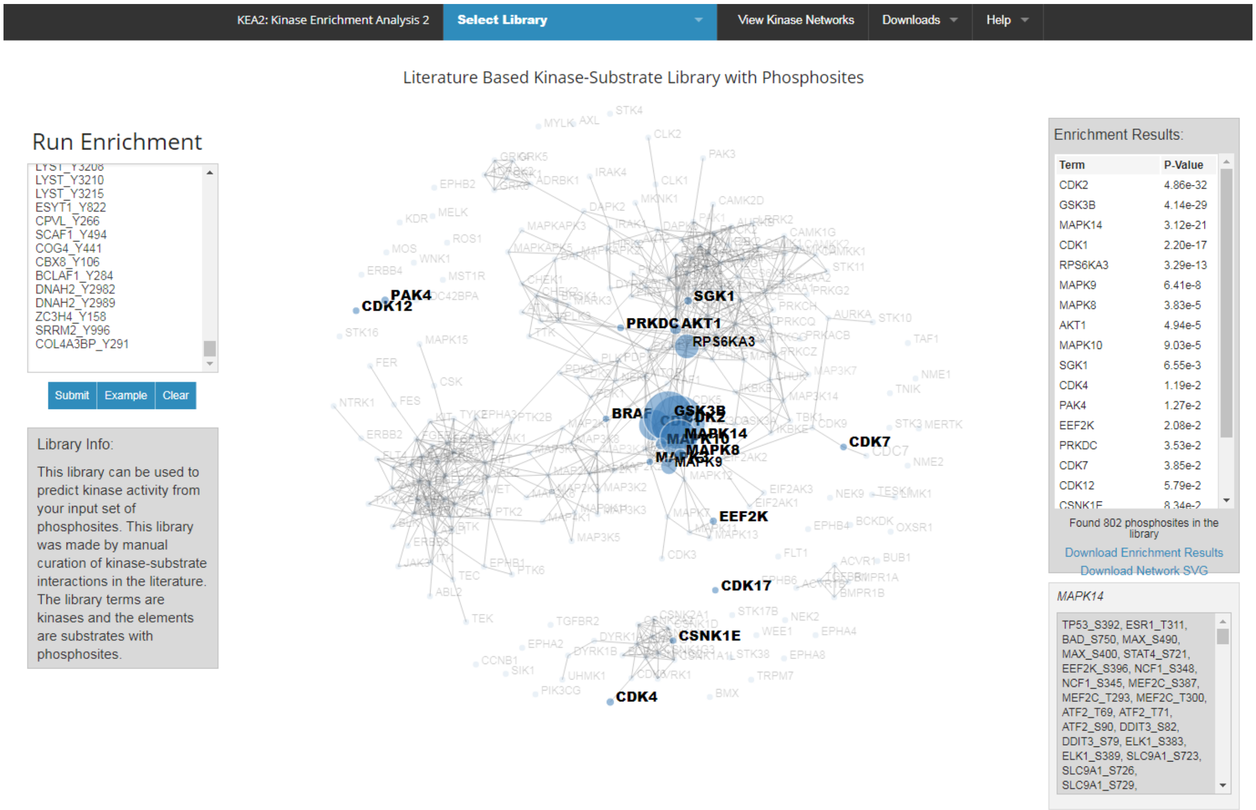

Figure S3, Related to Figure 2: Screenshot of the KEA2 output reporting kinase activity determined from Jurkat cell lysates subjected to high-pH reversed phase fractionation and subsequent phosphopeptide enrichment analyzed by shotgun LC-MS.

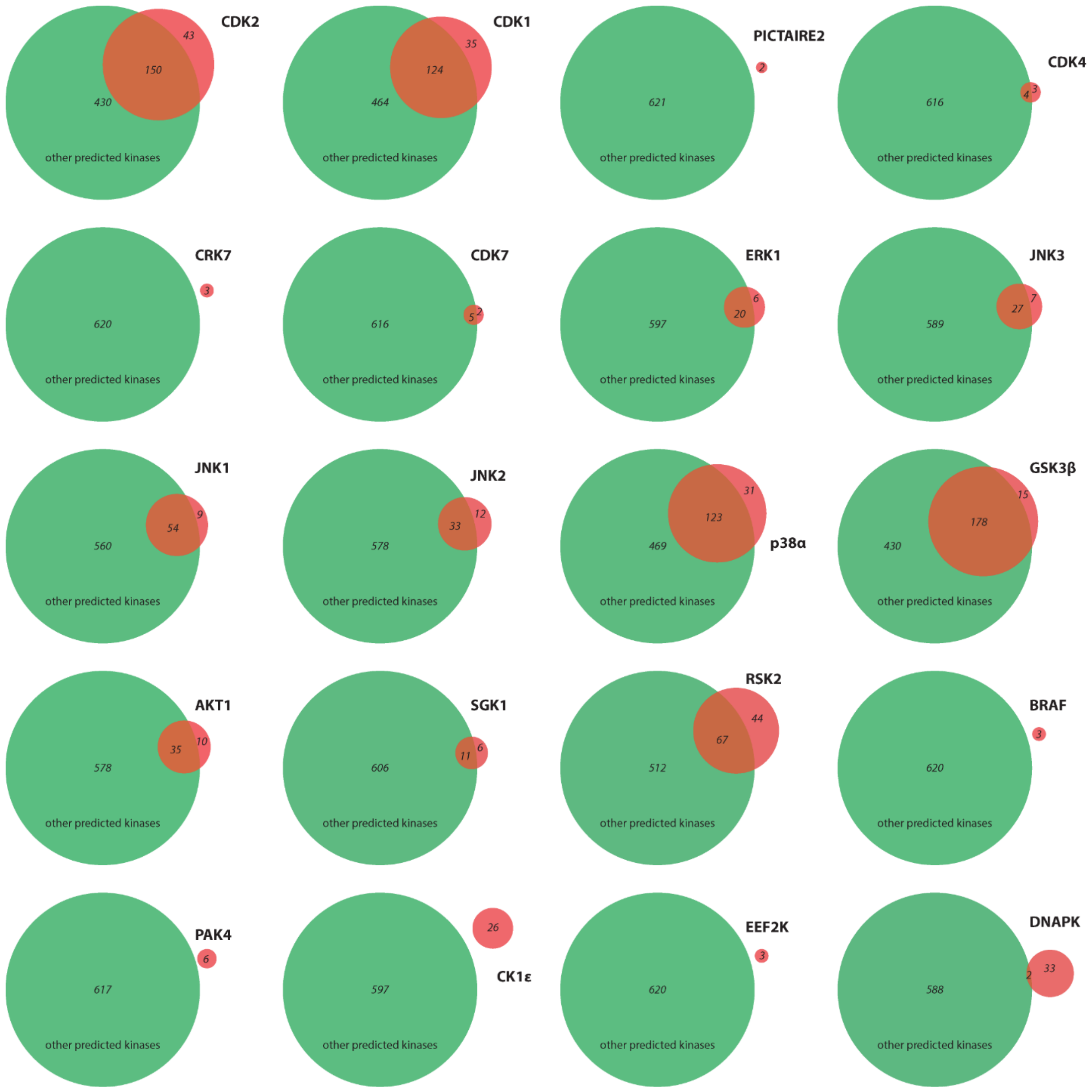

Figure S4, Related to Figure 2: Venn diagrams illustrating the amount of redundancy in kinase-substrate relationships impeding the kinase prediction by the online tool KEA2. Each Venn diagram compares the reported substrates to the reported substrates of the other predicted kinases.

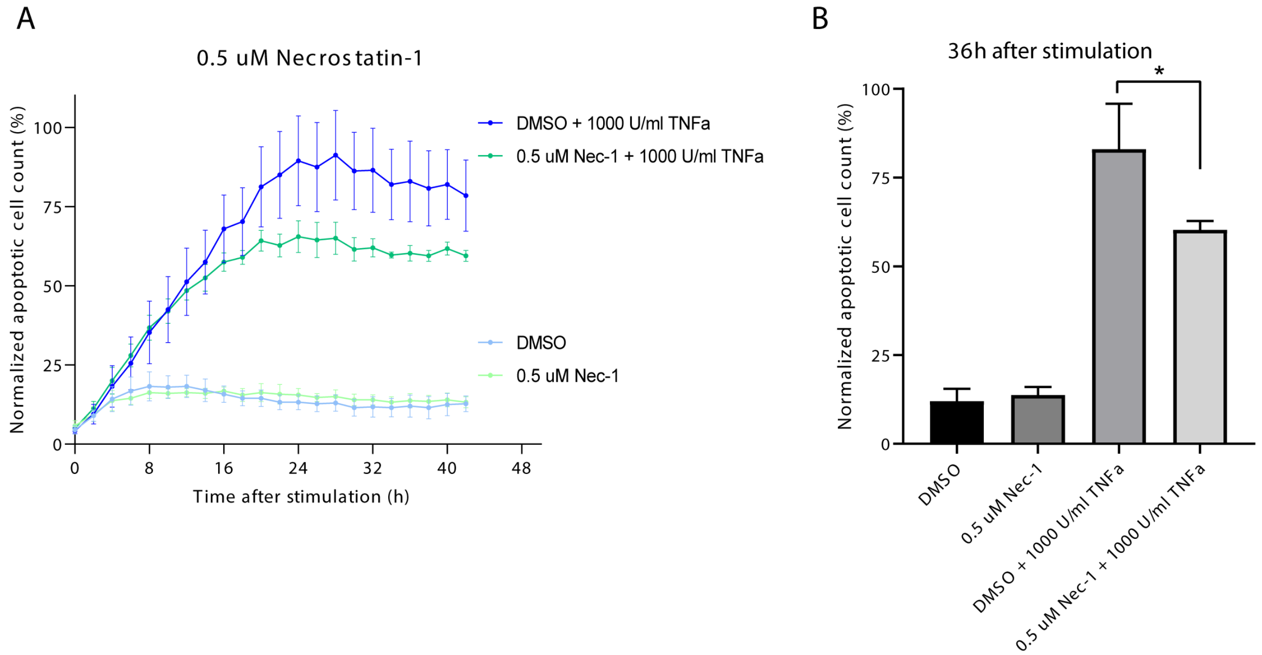

Figure S5, Related to Figure 2: Jurkat cell apoptosis upon TNFα treatment: Jurkat cells were grown with and without TNFα stimulation, in presence or absence of necrostatin-1 (Nec-1), and rate of cell death was determined by Caspase-3/7 green apoptosis reagent (n = 4 for all groups, error bars depicting standard deviation). (B) Apoptotic cell count after 36 hours in the different conditions showing a significant reduction in apoptotic cells after TNFα stimulation in the presence of Nec-1.
